# Cannabinoid-2 receptor depletion promotes non-alcoholic fatty liver disease in mice via disturbing gut microbiota and tryptophan metabolism

**DOI:** 10.1038/s41401-025-01495-w

**Published:** 2025-02-20

**Authors:** Wei-ting Cheng, Si-ya Pei, Jie Wu, Yan-jie Wang, Yong-wen Yang, Mei-fang Xiao, Jun Chen, Yuan-yuan Wang, Li Wu, Ze-bing Huang

**Affiliations:** 1 Department of Infectious Diseases, Xiangya Hospital, Central South University, Changsha, 410008 China; 2 Hunan Key Laboratory of Viral Hepatitis, Xiangya Hospital, Central South University, Changsha, 410008 China; 3 Nation Clinical Research Center for Geriatric Disorders, Xiangya Hospital, Central South University, Changsha, 410008 China; 4 Department of Blood Transfusion, Xiangya Hospital, Clinical Transfusion Research Center, Central South University, Changsha, 410007 China; 5 Shantou University Medical College, Shantou, 515041 China; 6 Department of Clinical Laboratory, Xiangya Hospital, Central South University, Changsha, 410008 China; 7 Department of Health Management Center, Xiangya Hospital, Central South University, Changsha, 410008 China

**Keywords:** non-alcoholic fatty liver disease, cannabinoid-2 receptor, gut microbiota, tryptophan metabolites

## Abstract

Non-alcoholic fatty liver disease (NAFLD) is the hepatic manifestation of the metabolic syndrome. NAFLD encompasses a spectrum of liver damage starting with liver steatosis and lipid disorders presented as the hallmark. Cannabinoid-2 receptor (CB2R) is the receptor of endocannabinoids mainly expressed in immune cells. Our preliminary study revealed the preventative role of CB2R in liver injury related to lipid metabolism. In this study, we aimed to explore the role of CB2R in NAFLD and the underlying mechanism related to microbial community. High-fat diet-induced NAFLD model was established in mice. We found that hepatic CB2R expression was significantly reduced in NAFLD mice and CB2R^–/–^ mice fed with normal chow. Interestingly, cohousing with or transplanted with microbiota from WT mice, or treatment with an antibiotic cocktail ameliorated the NAFLD phenotype of CB2R^–/–^ mice. The gut dysbiosis in CB2R^–/–^ mice including increased Actinobacteriota and decreased Bacteroidota was similar to that of NAFLD patients and NAFLD mice. Microbial functional analysis and metabolomics profiling revealed obviously disturbed tryptophan metabolism in NAFLD patients and NAFLD mice, which were also seen in CB2R^–/–^ mice. Correlation network showed that the disordered tryptophan metabolites such as indolelactic acid (ILA) and xanthurenic acid in CB2R^-/-^ mice were mediated by gut dysbiosis and related to NAFLD severity indicators. In vitro and in vivo validation experiments showed that the enriched tryptophan metabolites ILA aggravated NAFLD phenotypes. These results demonstrate the involvement of CB2R in NAFLD, which is related to gut microbiota-mediated tryptophan metabolites. Our findings highlight CB2R and the associated microbes and tryptophan metabolites as promising targets for the treatment of NAFLD.

## Introduction

Nonalcoholic fatty liver disease (NAFLD) is the hepatic manifestation of the metabolic syndrome. NAFLD encompasses a spectrum of liver damage starting with liver steatosis and lipid disorders presented as the hallmark [1, 2]. Nowadays, approximately 25% of the world’s population is living with NAFLD. The growing worldwide epidemic during the past decades led NAFLD to become the most rapidly growing contributor of liver mortality and morbidity [3–5]. And it has already been one of the most common etiologies for liver transplantation in the United States [6]. However, there still no approved therapies for NAFLD [1]. The unclear molecular mechanisms that cause the initiation and progression of NAFLD challenged the drug development.

Endocannabinoids are lipid mediators that interact with G protein-coupled receptors cannabinoid-1 receptor (CB1R) and cannabinoid-2 receptor (CB2R) [7]. CB1R is highly expressed in the central nervous system and much lower in peripheral tissues including the liver [8]. It was widely recognized that the endocannabinoid/CB1R system became overactive in metabolic syndrome including NAFLD, leading to increased food intake and decreased energy expenditure [9]. Thus, the CB1R antagonists were once being promising clinical candidates for treating metabolic disorders [10]. However, the psychiatric side effects halted the developing of CB1R blockers [9]. In contrast, CB2R is mainly expressed in peripheral tissues with immune functions and play critical roles in inflammatory process [11]. Our preliminary study revealed the preventive role of CB2R in liver injury [12]. Unexpectedly, the metabolomics analysis related it to lipid metabolism. These findings enlightened us that the CB2R might be involved in diseases featured by lipid disorders like NAFLD, spurring us to explore the underlying mechanism of CB2R in modulating lipid metabolism.

Over the past decades, the critical role of gut microbiota in NAFLD has been increasingly recognized. The impact of gut microbiota on lipid metabolism was extensively investigated [13]. And the modulating role of gut bacterial community on host lipid metabolism was relied on a wide variety of microbial metabolites [13]. Altered microbes were associated with liver steatosis in NAFLD patients [14, 15]. Gut microbiota and their metabolites controlled the absorption, emulsification, solubilization, synthesis, transportation and catabolism of lipids [16, 17]. Restoration microbial community by probiotics reversed the dyslipidemia and liver steatosis of NAFLD [18]. Due to the well-established role of gut microbiota in lipid metabolism, it is inevitable to explore the possible relationships between CB2R and gut dysbiosis in NAFLD development.

In this study, we aimed to reveal the role of CB2R in NAFLD using high-fat diet (HFD)-induced model mice and CB2R^-/-^ mice. Meanwhile, the potential correlation between CB2R and gut dysbiosis in NAFLD was investigated by structural and functional analysis based on 16S rRNA gene sequencing. Additionally, metabolomics approaches and correlation network were performed, and validation experiments were conducted to explore the underlying mechanism mediating the impact of CB2R on NAFLD development. Our study might provide novel insight for endocannabinoids system on NAFLD in the perspective of CB2R and suggest promising targets.

## Materials and methods

### Human samples

Serum and fecal samples for further 16S rRNA gene sequencing and untargeted metabolomics were obtained from the NAFLD patients who visited the liver clinic and were diagnosed with fatty liver disease at Xiangya Hospital during June 1, 2022 and June 1, 2023. Patients who were eligible for inclusion were aged 20–70 years and had a diagnosis of non-alcoholic steatohepatitis based on serum liver enzyme levels, imaging studies (B-ultrasonic examination or MRI scans) and Fibroscan [19]. The exclusion criteria were as follows: (1) excessive alcohol consumption (210 g/week in men or 140 g/week in women); (2) fatty liver suspected to be secondary to other liver diseases (chronic infection with Hepatitis B and/or C, autoimmune hepatitis and hepatolenticular degeneration) and other causes (hepatotoxic drugs, thyroid dysfunction, Inflammatory bowel disease, Cushing syndrome); (3) pregnant or chronic consumptive disease (malignant tumor, tuberculosis and HIV); (4) lack of relevant data or not cooperate [20]. In addition, 10 age- and sex-matched healthy controls were recruited and samples were collected. Detailed characteristics of patients with NAFLD are listed in Supplementary Table S1. All experiments were undertaken with the understanding and written consent of each participant. The clinical trial was registered in the NIH registration website (No. NCT05625750). The experiment procedures were approved by the Ethics Committee of the Central South University, Changsha, China.

### Animal experiments

C57BL/6 wild-type mice were purchased from the Department of Laboratory Animals, Central South University (Changsha, China). CB2R^-/-^ mice were obtained from Jackson Laboratories, USA (B6.129P2-Cnr2tmlDgen.005786). All of the above mice were housed in a specific pathogen-free facility with a standard controlled environment (20–25 °C, 50%–70% humidity, with natural lighting) and all of the animal protocols were approved by the Laboratory Animal Welfare Ethics Committee of Central South University, following the Health Guidelines of National Institutes for the Care and Use of Laboratory Animals (No. CSU-2023-0557).

After acclimated in one cage for one-week, male wild-type mice (6–12 weeks old, 18–30 g) were fed with HFD (contains 60% calories; D12492, Research Diets, New Brunswick, USA) accompanied with carbohydrates (18.9 g/L sucrose + 23.1 g/L fructose) in drinking water for 19 weeks to induce NAFLD [21], and the wild-type mice fed with normal control diet (NCD) as control. CB2R^–/–^ mice were fed with NCD.

Fresh food and water were supplied twice a week, and the body weight as well as food and water consumption were recorded weekly.

### Co-housing experiment, fecal microbiota transplantation and antibiotic treatment

For co-housing experiment, age-matched male CB2R^–/–^ and wild type (WT) mice (4–6 weeks old) were co-housed (up to 6 mice/cage) together in a 1:1 ratio for continuous 8 weeks [22].

For fecal microbiota transplantation, feces were collected from WT mice with NCD or HFD. 1200 mg fresh feces pallets were resuspended with a vortex in 4500 μL of phosphate buffered saline (PBS), and subsequently filtered with 70 μm cell strainer. CB2R^–/–^ mice were administered with 200 μL of the microbiota suspension by oral gavage once daily for 10 weeks. CB2R KO mice in the empty transplant group receive PBS.

For antibiotic treatment, an antibiotic cocktail was added to sterile water at a concentration of 1 g/L each for ampicillin, neomycin, and metronidazole and 500 mg/L for vancomycin [21]. This antibiotic-contained water was supplied as drinking water to CB2R^–/–^ mice for 4 weeks and was changed twice weekly. To assess microbiome depletion, fresh fecal samples were collected at 4 weeks post-treatment and frozen at –80 °C before use. Commercial stool DNA extraction kit (EK1212, ECOTOP, Guangzhou, China) was used for fecal bacteria quantification as recommended by the manufacturers. More details are provided in the Supplementary Information.

### Tryptophan metabolites treatment

Mouse hepatocyte cell line AML12 were cultured in specialized medium (CM0602, Procell, WuHan, China) at 37 °C in humid air with 5% CO_2_. Free fatty acid (FFA, PA/OA/BSA conjugates) was prepared as primary described to induce hepatocyte steatosis, with a final PA concentration of 0.33 mM and a final OA concentration of 0.66 mM. ILA (I157602, Aladdin, Shanghai, China) and xanthurenic acid (Xa, S4774, Selleck, Houston, TX, USA) were dissolved in DMSO. AML12 cells were pretreated with DMSO, ILA (100 μM) or Xa (100 μM) for 12 h, subsequently stimulated with BSA or FFA conjugates for another 24 h.

Indole lactic acid (ILA) was used for in vivo experiments, and male C57BL/6 J mice (6-weeks) were randomly divided into NCD group and HFD group for eight weeks. Then the mice in HFD group were randomly redivided into two groups (*n* = 3 per group) : (1) ILA: mice were administered ILA (I157602, Aladdin, Shanghai, China) dose at 40 mg/kg orally once daily (8 mg/mL in sterile water). (2) HFD: mice were given oral gavage with sterile water at dose of 5 μL/g. Meanwhile, normal chow mice were given oral gavage with an equal volume of sterile water.

### Measurement of biochemical parameters

Serum was collected from mice orbital blood after experiment completed. Alanine aminotransferase (ALT), aspartate aminotransferase (AST), triglyceride (TG) and total cholesterol (TC) levels were measured using reagent kits (Nanjing Jiancheng Bioengineering Institute, Nanjing, China) according to manufacturer’s instructions [23, 24]. Blood glucose was measured by an Accu-Chek glucometer (Yuwell, Nanjing, China). For hepatic lipid profile, a piece of liver tissue was homogenized in anhydrous ethanol and TG content was quantified with commercial kit (Nanjing Jiancheng Bioengineering Institute, Nanjing, China) [25].

### Histological and immunohistochemical examination of the liver

Immediately after sacrifice of the mice, liver and intestine samples were submerged in 4% paraformaldehyde overnight and subsequently embedded in paraffin wax, sectioned at 5 μm. Then the liver and intestine sections were stained with hematoxylin and eosin (H&E) for histopathological analysis or a CB2R primary antibody (1:100; ab3559; Abcam, Cambridge, MA, USA) for immunohistochemistry (IHC). For Oil Red O staining, frozen liver specimens embedded in Tissue-Tek OCT compound (4583; SAKURA, Torrance, CA, USA) were sectioned at 20 μm, and then stained with Oil Red O to evaluate lipid droplet accumulation [26].

### The bacterial 16S rRNA gene sequencing

Fresh fecal samples were collected into the sterile cryogenic microtubes and stored at –80 °C until use. DNA extractions from fecal pellets were performed using Magnetic Stool DNA Kit (TianGen, China) in accordance with the manufacturer’s instruction. Then, the hypervariable V3-V4 region of bacterial 16S rRNA gene was amplified by PCR using forward primer (5ʹ-CCTAYGGGRBGCASCAG-3ʹ) and reverse primer (5ʹ-GGACTACNNGGGTATCTAAT-3ʹ). PCR reactions were purified and quantified. Next, the equimolar PCR reactions were pooled and sequenced using the Illumina NovaSeq PE250 high-throughput sequencing platform (San Diego, CA, USA).

All reads with 97% similarity were clustered into operational taxonomic units (OTUs), and OTU abundance tables were generated with multiple sequence alignment in QIIME2 analysis systems [27]. For α-diversity, including diversity indices (Chao1 index, observed_features) and species richness (Shannon index, Simpson index), unpaired *t* test or Mann–whitney test for two group, and one-way ANOVA test or Kruskal-Wallis test for more than two groups were used to determine statistically significance. The PERMANOVA was applied to assess β-diversity of the community structure differentiation (principal coordinates analysis (PCoA) based on weighted UniFrac distance matrices and unweighted UniFrac distance matrices). The linear discriminant analysis effect size (LEfSe) method was used to identify distinguishing microbes with LDA score > 2 as threshold. Taxonomic comparisons were analyzed with multiple *t* tests. Then, a functional profile of KEGG Orthology (KO) for each sample was predicted from 16S rRNA information with PICRUSt2 and statistical analysis was conducted by STAMP software (v2.1.3) [28]. Enrichment analysis with the significant KO profile was performed in MicrobiomeAnalyst program (https://www.microbiomeanalyst.ca/) and visualized with enrichment dot bubble in Novogene (https://magic.novogene.com/).

### High-throughput untargeted metabolomics profiling

#### Sample preparation

The blood sample was centrifuged at 3000 rpm for 15 min at room temperature, and the serum was collected and preserved at –80 °C for metabolome analysis. An equal volume of serum sample (50 μL) was mixed with 200 μL methanol spiked with an isotopically-labeled internal standard mixture, vortexed for 30 s, sonicated in ice-water bath for 10 min, and then all samples were set aside at –40 °C for 1 h [29]. After centrifugation at 12,000 rpm for 15 min at 4 °C, the supernatant was transferred to a new glass vial for processing. The quality control (QC) sample was prepared by combining an equal aliquot of each sample to assess the consistency of the assay system and method.

#### Untargeted metabolomics profiling

LC-MS/MS analysis was performed by an UHPLC system (Vanquish, Thermo Fisher Scientific) with a UPLC HSS T3 column (2.1 mm × 100 mm, 1.8 μm) coupled to an Orbitrap Exploris 120 mass spectrometer (Orbitrap MS, Thermo Fisher Scientific). Phase A was composed of 5 mmol/L ammonium acetate and 5 mmol/L acetic acid in water, and phase B was acetonitrile. Sample plate temperature was maintained at 4 °C, and the injection volume was 2 μL. The Orbitrap Exploris 120 mass spectrometer was used to capture MS/MS spectra in an information-dependent acquisition (IDA) mode under the control of Xcalibur acquisition software (v4.4, Thermo Fisher Scientific). The electrospray ionization (ESI) source conditions were set as following: sheath gas flow rate as 50 Arb, aux gas flow rate as 15 Arb, capillary temperature as 320 °C, full MS resolution as 60,000, MS/MS resolution as 15,000, collision energy as 10/30/60 in NCE mode, and spray voltage as 3.8 kV (positive) or -3.4 kV (negative).

#### Data analysis

The raw data were transformed into mzXML format using ProteoWizard software and processed for pretreatments including peak detection, extraction, alignment, and integration using R based on XCMS [30]. An in-house-generated MS2 database (BiotreeDB) was applied for metabolite annotation. The threshold for annotation was set at 0.3.

Principal component analysis (PCA) and orthogonal projections to latent structures-discriminant analysis (OPLS-DA) were used to identify the overall distributive tendencies and the degree of difference in the metabolic profiles among groups by SIMCA software (v16.0.2, Sartorius Stedim Data Analytics AB, Umea, Sweden) [31]. Variable importance prediction (VIP) of OPLS-DA lower than 1 and *P* value of Student’s *t* test greater than 0.05 were removed and then left are typically identified as differential metabolites. Enrichment analysis was performed by MicrobiomeAnalyst program and the enrichment pathway was visualized by Novogene. Heatmap was drawn to determine the characteristic changes of the differential metabolites between groups by Cluster 3.0 software.

### RNA-sequencing (RNA-seq) analysis of liver

Liver tissues were washed with RNase free water and flash-frozen in liquid nitrogen. Total RNA was extracted with the TRIzol reagent (Invitrogen Life Technologies, Carlsbad, CA, USA), the purity of RNA was detected with Thermo Scientific NanoDrop 2000 (Thermo Fisher Scientific, Waltham, MA, USA) and the integrity of RNA was assessed with Agilent 2100 Bioanalyzer (Agilent Technologies, PaloAlto, CA, USA). Subsequently, the sequencing library was constructed with NEBNext Ultra II RNA Library Prep Kit for Illumina and further sequenced on NovaSeq 6000 platform (Illumina, San Diego, CA, USA). Four biological repeats were performed in each group. The sequencing data were filtered using fastp software (v0.22.0) to get high-quality reads. HISAT2 software (v2.1.0) was applied for mapping the reads to the reference genome. Gene expression was measured by HTseq (v0.9.1) and standardized using Fragments Per Kilo bases per Million fragments (FPKM) / Transcripts per Million (TPM) for further analysis. Absolute fold change larger than 1 and *P* value less than 0.05 was considered as significant differential expressed genes (DEGs) analyzed by DESeq2 (v1.38.3). ClusterProfiler (v4.6.0) was used to carry out GO and KEGG enrichment analysis as well as gene set enrichment analyses (GSEA).

### Statistical analysis

All data are presented as mean ± SEM or median with range. Statistical analysis (Student’s *t* test or Mann-whitney test for two group and one-way ANOVA test or Kruskal-Wallis test for more than two groups) and bar chart plotting were performed using GraphPad Prism 10.0 (Inc., La Jolla, CA, USA), and *P* < 0.05 was considered significant. The correlation analysis was conducted by the Pearson’s or Spearman rank correlation analysis. The correlation coefficients with *P* < 0.05 were used to build a correlation network by Cytoscape software package and correlation heatmap was plotted by http://www.bioinformatics.com.cn/.

## Results

### The decreased expression of hepatic CB2R in HFD-induced NAFLD mice

**Fig. 1:**
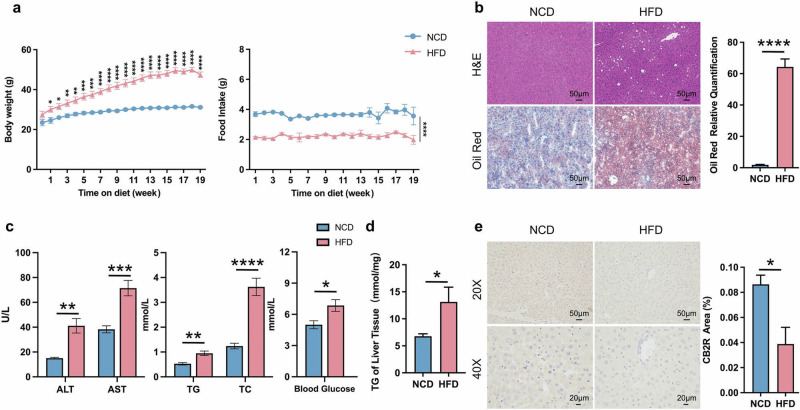
The decreased expression of hepatic CB2R in HFD induced NAFLD mice. **a** The body weight and food intake of WT mice with NCD or HFD throughout the experimental period were recorded. **b** The Representative images (200×) of H&E or Oil red O staining, and the morphometric analysis of Oil red O staining for quantification. **c** The comparison of serum levels of transaminases (ALT, AST) and lipid parameters (TG, TC), and blood glucose between WT mice with NCD or HFD. **d** The comparison of liver homogenate levels of TG. **e** Immunohistochemically labeled with CB2R of mice liver sections and quantitative analysis (200×, 400×). Note: Data were given as mean ± SEM. *n*: (**a**), *n* = 8 per group; (**b**), *n* = 4 per group; (**c**, **d**), *n* = 6 per group; (**e**), *n* = 3 per group. *P < 0.05; **P < 0.01; ***P < 0.001; ****P < 0.0001. NCD normal control diet, HFD high fat diet, WT wild type, CB2R cannabinoid-2 receptor, NAFLD nonalcoholic fatty liver disease, H&E hematoxylin & eosin, ALT alanine transaminase, AST aspartate transferase, TC total cholesterol, TG triglyceride, SEM standard error of mean.

The HFD was used to induce NAFLD mice. As shown in Fig. 1a and Supplementary Fig. S1a, b, although the food intake was obviously decreased, the body weight of wild type (WT) mice feeding with HFD was significantly increased when compared to those with normal chow diet (NCD) since the first week. 19-week HFD diet induced NAFLD in WT mice was evidenced by remarkably hepatic steatosis by H&E or Oil red O staining (Fig. 1b, Supplementary Fig. S1c). The serum transaminases including ALT and AST reflecting liver injury were also significantly increased in WT-HFD mice compared with WT-NCD mice (Fig. 1c, Supplementary Fig. S1e). And the WT-HFD mice also exhibited metabolic disorders indicated by increased serum lipid parameters and blood glucose as well as TG accumulation of liver tissue (Fig. 1c, d, Supplementary Fig. S1d, e). Notably, the expression of CB2R in liver was significantly decreased in HFD-induced NAFLD mice, while showed increased in intestine (Fig. 1e, Supplementary Fig. S2b, c). These findings suggested that the depletion of hepatic CB2R was related to the pathogenesis of NAFLD.

### The CB2R^–/–^ mice with NCD developed NAFLD

**Fig. 2:**
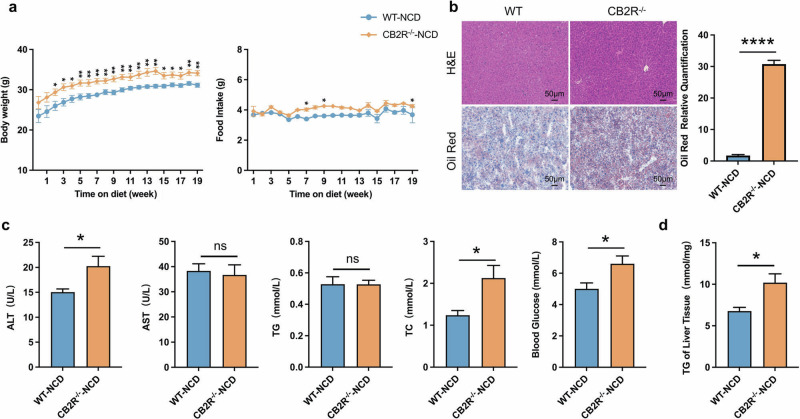
The CB2R^–/–^ mice with NCD developed NAFLD. **a** The body weight and food intake of WT mice with NCD or CB2R^-/-^ mice with NCD throughout the experimental period were recorded. **b** The Representative images (200×) of H&E or Oil red O staining, and the morphometric analysis of Oil red O staining for quantification. **c** The comparison of serum levels of transaminases (ALT, AST) and lipid parameters (TG, TC), and blood glucose between WT mice with NCD or CB2R^-/-^ mice with NCD. **d** The comparison of liver homogenate levels of TG. Note: Data were given as mean ± SEM. *n*: (**a**), WT-NCD, *n* = 8 and CB2R^–/–^-NCD, *n* = 7; (**b**), *n* = 4 per group; (**c**, **d**), *n* = 6 per group. *P < 0.05; **P < 0.01; ****P < 0.0001.

In order to demonstrate the critical role of CB2R in the pathogenesis of NAFLD, CB2R^–/–^ mice were applied (Supplementary Fig. S3). The expression of CB2R in CB2R^-/-^ mice was remarkably decreased in all tissues especially the liver (Supplementary Fig. S2a). As shown in Fig. 2a and Supplementary Fig. S1a, b, the body weight of CB2R^-/-^-NCD mice was significantly increased when compared to WT-NCD mice, while the food and water intake were unchanged. Similar to HFD-induced NAFLD mice, the H&E or Oil red O staining results showed that CB2R^–/–^ mice with NCD presented with steatosis in the liver (Fig. 2b, Supplementary Fig. S1c). Meanwhile, the serum ALT, TC levels, and the blood glucose level as well as hepatic TG content were higher in CB2R^-/-^ mice with NCD compared with the NCD-feeding WT mice (Fig. 2c, d, Supplementary Fig. S1d, e).

To further assess the impact of CB2R on lipid metabolism, we performed hepatic RNA sequencing of CB2R^–/–^ mice. The volcano plots and clustering heat map presented the distinct hepatic gene expression of CB2R^–/–^ mice from WT mice (Supplementary Fig. S4a, b). The KEGG and GO pathway enrichment analysis exhibited the differential pathways in the liver including cell communication, cell death, cytokines (TGF-beta, IL-17) and inflammatory process like NF-kappa B signaling between CB2R^–/–^ mice and WT mice (Supplementary Fig. S4c, d). Moreover, GSEA analysis based on GO modules was conducted to reveal the distinctive lipid metabolism of CB2R^–/–^ mice. As shown in Supplementary Fig. S4e–g, we found that lipid metabolism-associated gene sets, including fatty acid biosynthetic process (NES = 0.82, *P* value = 0.928) and fatty acid oxidation (NES = –0.73, *P* value = 0.964) had no significant difference between CB2R^-/-^ mice and WT mice, while fatty acid transport (NES = –1.43, *P* value = 0.029) were significantly decreased in CB2R^–/–^ mice. Furthermore, analysis of genes implicated in major aspects of lipid metabolism revealed that depletion of CB2R upregulated the transcription of DNL-associated gene SREBF1 and lipid export-associated gene LDLR but did not affect the transcription of lipid uptake, oxidation-related genes (Supplementary Fig. S4h) [32, 33]. These findings suggested that CB2R deficiency promote NAFLD via disturbing lipid metabolism including lipid biosynthesis and export.

### Co-housing with or transplanted microbiota from WT mice and antibiotic treatment ameliorated the NAFLD phenotype of CB2R^–/–^ mice

**Fig. 3:**
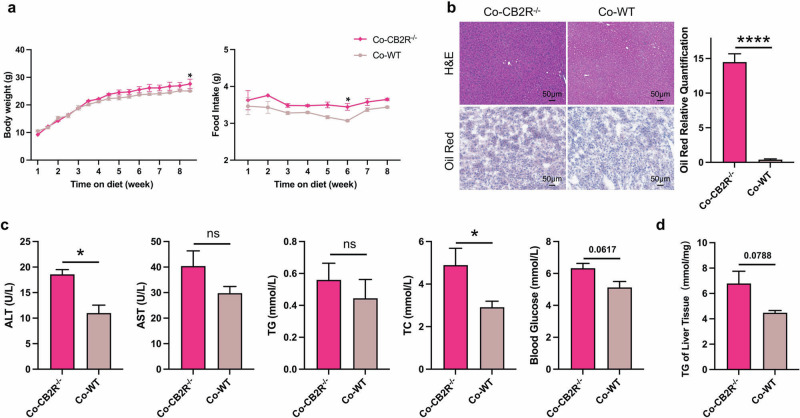
The severity of NAFLD in CB2R^–/–^ mice was improved when cohousing with WT mice. **a** The body weight and food intake of CB2R^–/–^ mice cohousing with WT mice or CB2R^–/–^ mice throughout the experimental period were recorded. **b** The representative images (200×) of H&E or Oil red O staining, and the morphometric analysis of Oil red O staining for quantification. **c** The comparison of serum levels of transaminases (ALT, AST) and lipid parameters (TG, TC), and blood glucose between CB2R^-/-^ mice cohousing with WT mice or CB2R^–/–^ mice. **d** The comparison of liver homogenate levels of TG. Two experiments were repeated independently with similar results in Fig. 3 and Fig. S5. Note: Data were given as mean ± SEM. *n*: (**a**–**d**), *n* = 3 per group. *P < 0.05; ****P < 0.0001. Co cohousing.

In order to explore the potential mechanism of CB2R in disturbing hepatic lipid metabolism, we further investigated the contribution of microbiota which is known to be the key modulator of hepatic steatosis in NAFLD [34]. A “cohousing” assay was performed as primary described [35]. We co-housed CB2R^-/-^ mice (4–6 weeks old) with WT or CB2R^–/–^ mice in same cage for eight weeks. As shown in Fig. 3a and Supplementary Fig. S5a, the food and water intake was almost unchanged while the body weight of CB2R^–/–^ mice was significantly decreased when cohousing with WT mice compared to those still with CB2R^–/–^ mice. Meanwhile, hepatic steatosis as indicated by H&E or Oil red O staining was also improved in the CB2R^–/–^ mice cohousing with WT mice (Fig. 3b, Supplementary Fig. S5b). Moreover, the level of parameters indicating metabolic disorders including TC and blood glucose as well as ALT were lower in CB2R^–/–^ mice cohousing with WT mice (Fig. 3c, Supplementary Fig. S5c). And CB2R^-/-^ mice cohousing with WT mice showed decreased TG accumulation in liver (Fig. 3d, Supplementary Fig. S5d). Strikingly, WT mice co-housed with CB2R^–/–^ mice appeared NAFLD phenotype, with increased body weight, hepatic steatosis indicated by H&E and Oil red O staining as well as higher serum levels of ALT, TC and liver TG content (Supplementary Fig. S6a–d).

**Fig. 4:**
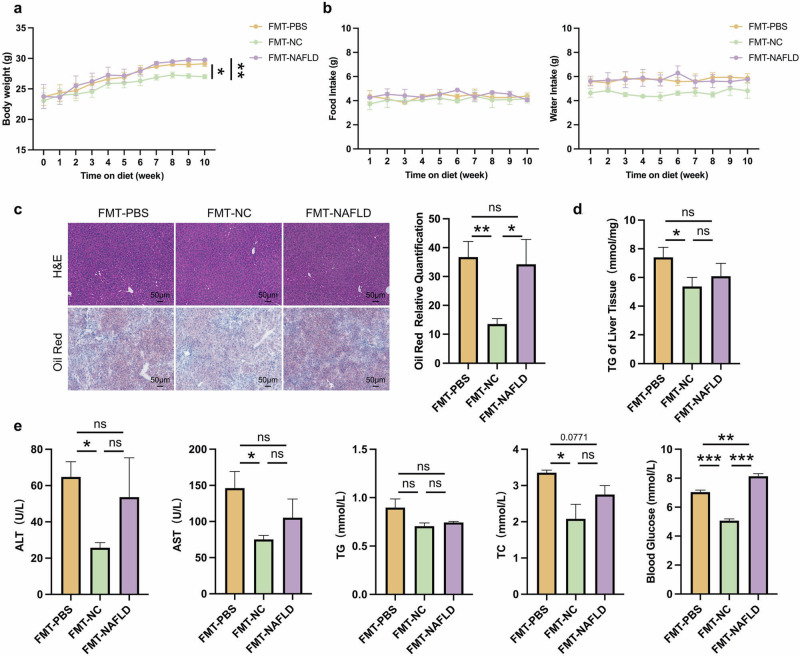
The severity of NAFLD in CB2R^-/-^ mice was alleviated when transplanted with WT NCD mice gut microbiota. **a** The body weight of CB2R^-/-^ mice transplanted with PBS or gut microbiota of WT mice with NCD or WT NAFLD model mice throughout the experimental period were recorded. **b** The food intake and water intake. **c** The representative images (200×) of H&E or Oil red O staining, and the morphometric analysis of Oil red O staining for quantification. **d** The comparison of liver homogenete levels of TG. **e** The comparison of serum levels of transaminases (ALT, AST) and lipid parameters (TG, TC), and blood glucose between CB2R^–/–^ mice transplanted with PBS or gut microbiota of WT mice with NCD or WT NAFLD model mice. Note: Data were given as mean ± SEM. *n*: (**a**), *n* = 4 per group; (**b**–**e**), *n* = 3 per group. *P < 0.05; **P < 0.01; ***P < 0.001; ns, no significance. FMT fecal microbiota transplantation, NC normal control, PBS phosphate buffered saline.

Based on the aforementioned results, we performed fecal microbiota transplantation (FMT) to confirm the relationship between microbiota and NAFLD phenotype of CB2R^–/–^ mice [35]. We use WT mice with NCD or HFD as microbiota donors and CB2R^–/–^ mice as recipients. As shown in Fig. 4a, CB2R^–/–^ mice exhibited a loss of body weight when transplanted with microbiota from WT mice compared to CB2R^–/–^ mice transplanted with PBS, and the food intake and water intake were unchanged. Histologically, H&E and Oil red O staining revealed fewer hepatic steatosis in the CB2R^–/–^ mice transplanted with microbiota from WT mice (Fig. 4c). Accordingly, the levels of serum TC and blood glucose as well as hepatic TG content was significantly lower in CB2R^–/–^ mice transplanted with microbiota from WT mice compared to CB2R^-/-^ mice transplanted with PBS (Fig. 4d, e). Furthermore, the degree of liver injury, as measured by serum levels of ALT and AST in CB2R^–/–^ mice transplanted with microbiota from WT mice group, was significantly lower than that in the vehicle group (Fig. 4e). Of note, CB2R^-/-^ mice transplanted with microbiota from NAFLD model mice indicated an increase in blood glucose compared to CB2R^-/-^ mice transplanted with PBS while had no significant difference in body weight, hepatic steatosis, serum parameters and liver TG accumulation (Fig. 4a–e).

**Fig. 5:**
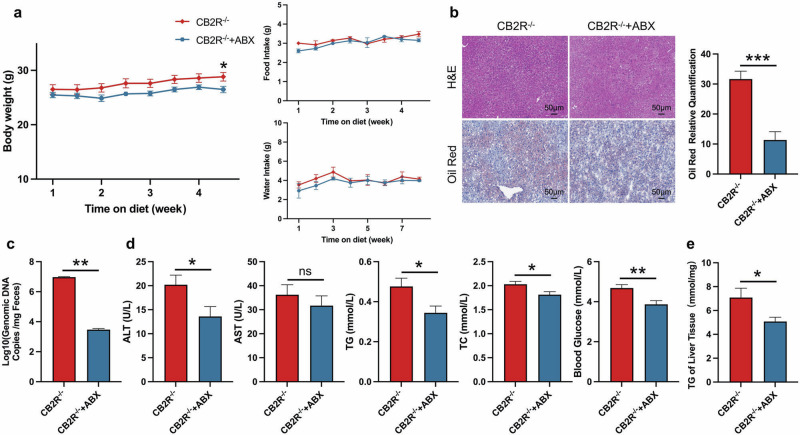
Antibiotic treatment alleviates the NAFLD phenotype of CB2R^-/-^ mice. **a** The body weight, food intake and water intake of CB2R^-/-^ mice treated with ABX or sterile water throughout the experimental period were recorded. **b** The representative images (200×) of H&E or Oil red O staining, and the morphometric analysis of Oil red O staining for quantification. **c** qRT-PCR analysis of bacteria genomic copies in feces from CB2R^–/–^ mice treated with antibiotic or sterile water. **d** The comparison of serum levels of transaminases (ALT, AST) and lipid parameters (TG, TC), and blood glucose between CB2R^–/–^ mice treated with ABX or sterile water. **e** The comparison of liver homogenate levels of TG. Note: Data were given as mean ± SEM. *n*: (**a**–**e**), *n* = 6 per group. *P < 0.05; **P < 0.01; ***P < 0.001; ns no significance. ABX antibiotic.

To further verify the hypothesis that CB2R depletion contributes to NAFLD via disrupting gut microbiota, we treated CB2R^-/-^ mice with broad-spectrum antibiotics (ABX) for four weeks to deplete the intestinal microbiota. The ABX-treated mice had a sharp decrease in commensal microbes as indicated by total bacterial DNA (Fig. 5c). More importantly, compared to vehicle controls, ABX-treated mice exhibited a similar alleviation of NAFLD as CB2R^–/–^ mice co-housed with or transplanted with microbiota from WT mice, as indicated by reduced body weight, lower serum levels of TG, TC and blood glucose, alleviated hepatic steatosis and reduced hepatic TG level (Fig. 5a, b, d, e). These findings manifested that the gut microbiota changes play important roles in NAFLD development of CB2R^–/–^ mice.

### Alterations of gut microbiota in NAFLD patients

**Fig. 6:**
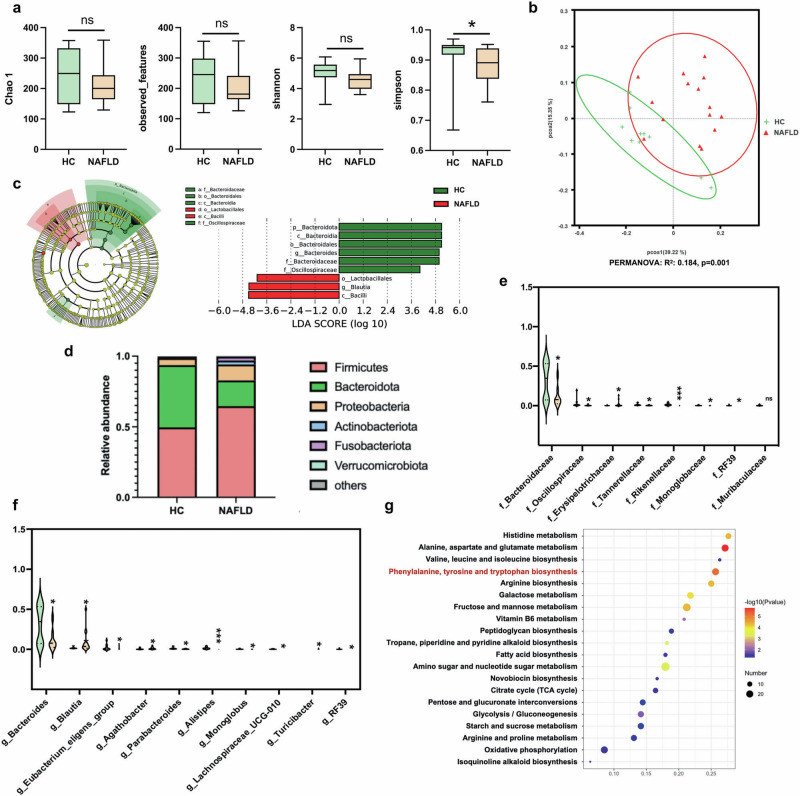
Altered gut microbiota in NAFLD patients. **a** The microbial richness and diversity in feces of study subjects were depicted according to α-diversity parameters including Chao 1, observed_feature and Shannon and Simpson index. **b** The β-diversity between HC and NAFLD patients was presented by PCoA plots. **c** LEfSe analysis and the cladogram diagram showed the microbes with significant differences between HC and NAFLD patients; (**d**) the differential microbial structure at phylum level between HC and NAFLD patients; Comparison of microbes at (**e**) family and (**f**) genus levels; (**g**) the pathway enrichment analysis was performed based on the predicted functional composition of microbial community. Note: Data were given as median with range. *n*: (**a**–**g**), HC, *n* = 10 and NAFLD, *n* = 15. *P < 0.05; ***P < 0.001 compared with HC. HC healthy control, PCoA principal coordinates analysis, LEfSe the linear discriminant analysis effect size.

Since the gut microbiota were known to play key roles in NAFLD pathogenesis, we further explored the association between CB2R depletion and gut dysbiosis in NAFLD and discovered the bacterial taxa might be of relevance to humans. Characteristics of 15 NAFLD patients and 10 health control (HC) individuals were summarized in Supplementary Table S1. As shown, there was no significant difference among NAFLD and HC group with respect to age, sex, and body height. In addition, there was also no difference in laboratory tests for AST, LDL cholesterol, total cholesterol, blood glucose and uric acid among two groups. Of note, there was significant differences in terms of body weight (*P* = 0.009), BMI (*P* < 0.0001). Moreover, NAFLD patients had significantly higher level of ALT (*P* = 0.026) and triglycerides (*P* = 0.004) as well as lower HDL cholesterol (*P* = 0.001). We collected the fresh fecal samples for 16S rRNA sequencing. As exhibited in Fig. 6a, although the alpha diversity index including Chao 1, observed_features and Shannon index were unchanged in NAFLD patients when compared with HC, the Simpson index reflecting the microbial diversity was obviously decreased in individuals with NAFLD. The beta diversity of the intestinal microecology between NAFLD and HC was exhibited by PCoA plot based on weighted UniFrac, which showed differential microbial structure under NAFLD status (PERMANOVA, *R*^2^ = 0.184, *P* = 0.001) (Fig. 6b). LEfSe analysis showed that the microbial features of NAFLD patients were characterized by decreased microbes in Bacteroidota phyla including Bacteroidota, *Bacteroidia*, *Bacteroidales*, *Bacteroidaceae* (Fig. 6c). Although *Oscillospiraceae* from Firmicutes was decreased, *Lactobacillales*, *Blautia* and *Bacilli* from Firmicutes were increased in NAFLD patients (Fig. 6c).

We further analyzed the predominant microbes with the relative abundance more than 1% in any of the groups. As shown in Fig. 6d, the major phyla in study subjects in HC or NAFLD groups were Firmicutes, Bacteroidota, Proteobacteria, Actinobacteriota, Fusobacteriota and Verrucomicrobiota. The ratio of Firmicutes, Proteobacteria, Actinobacteriota and Fusobacteriota were increased while Bacteroidota were decreased in NAFLD patients compared with HC. In terms of microbes at family level, *Erysipelotrichaceae* from Firmicutes was increased while *Oscillospiraceae, RF39* and *Monoglobaceae* from the same phyla was decreased in NAFLD patients (Fig. 6e). Consistent with the findings at phylum level, *Bacteroidaceae*, *Tannerellaceae* and *Rikenellaceae* from phyla Bacteroidota were decreased in NAFLD patients compared with HC (Fig. 6e). Similar to findings at phylum level, three genera including *Blautia*, *Agathobacter* and *Turicibacter* from Firmicutes were enriched, while *Bacteroides*, *Parabacteroides* and *Alistipes* from Bacteroidota were depleted in NAFLD patients compared with HC (Fig. 6f). The relative abundance of genus *RF39*, *Eubacterium_eligens_group*, *Monoglobus and Lachnoapiracea_UCG-010* were lower in NAFLD individuals than HC (Fig. 6f). The KEGG functional orthologs of microbial community were predicted using PICRUSt2 based on the 16S rRNA sequencing data. As shown in Fig. 6g, the gut microbiota in NAFLD showed functional changes including amino acid metabolism of histidine, alanine, aspartate, glutamate, valine, leucine, isoleucine, phenylalanine, tyrosine, tryptophan and arginine. The microbial function of carbohydrate metabolism including galactose, fructose, mannose, peptidoglycan, amino sugar and nucleotide sugar, starch and sucrose metabolism, as well as citrate cycle, pentose and glucuronate interconversions, glycolysis/gluconeogenesis were also shifted in NAFLD patients. Meanwhile, the fatty acid biosynthesis, vitamin B6 metabolism, tropane, piperidine and pyridine alkaloid synthesis, novobiocin biosynthesis, oxidative phosphorylation, isoquinoline alkaloid biosynthesis were also changed in microbial community of patients with NAFLD. These findings in study subjects suggested that gut dysbiosis was involved in NAFLD development.

### The microbial community of CB2R^–/–^ mice with NCD was similar to HFD-induced NAFLD mice

**Fig. 7:**
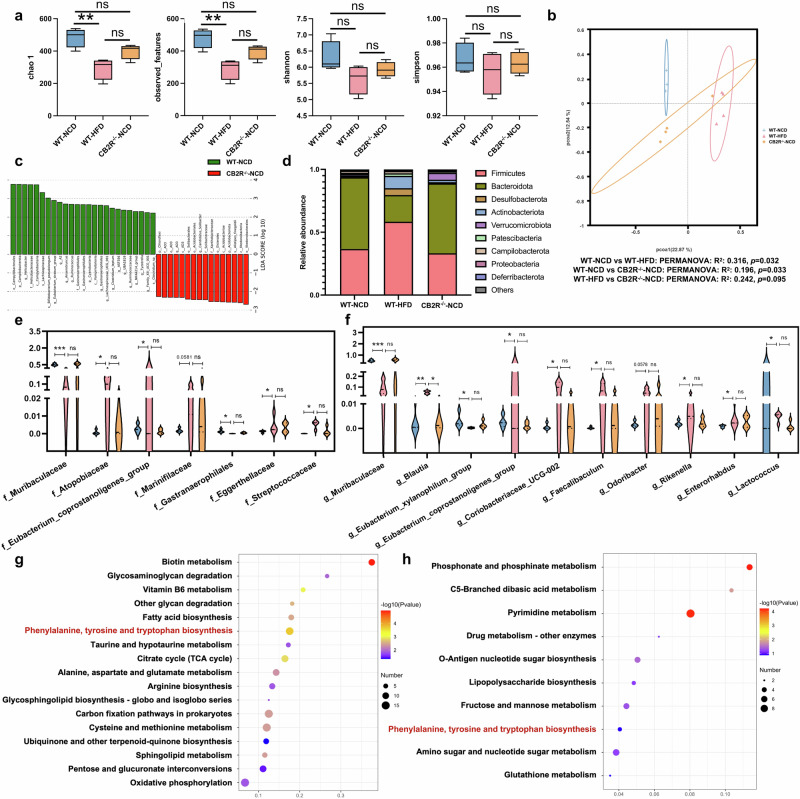
The microbial community of CB2R^-/-^ mice with NCD was similar to HFD-induced NAFLD mice. **a** The microbial richness and diversity in feces of mice were depicted according to α-diversity parameters including Chao 1, observed_feature and Shannon and Simpson index. **b** The β-diversity was presented by PCoA plots. **c** LEfSe analysis showed the microbes with significant differences between WT mice with CB2R^-/-^ mice with NCD. **d** The differential microbial structure at phylum level among three groups; Comparison of microbes at (**e**) family and (**f**) genus levels. The pathway enrichment analysis between WT mice feeding with NCD with (**g**) WT mice feeding with HFD or (**h**) CB2R^–/–^ mice feeding with NCD was performed based on the predicted functional composition of microbial community. Note: Data were given as median with range. *n*: (**a**–**h**), *n* = 4 per group. *P < 0.05; **P < 0.01 and ***P < 0.001.

In order to explore the association between CB2R depletion and gut dysbiosis in NAFLD, the microbial structure in CB2R^–/–^ mice and WT mice with NCD or HFD-induced NAFLD was analyzed. As exhibited in Fig. 7a, the Chao1 and observed_features reflecting microbial richness were decreased in WT NAFLD model mice compared with WT mice with NCD. Similar to findings in NAFLD patients, the Shannon and Simpson indexes showed decreased trend in NAFLD mice compared to WT mice feeding NCD (Fig. 7a). Interestingly, there was no significant difference in Chao1 and observed_features between CB2R^–/–^ mice and WT mice with NCD. To resemble NAFLD mice, the Shannon and Simpson indexes tended to decrease in CB2R^–/–^ mice with NCD compared to WT mice with NCD (Fig. 7a). The PCoA plots showed that the microbial community of NAFLD mice (PERMANOVA, *R*^2^ = 0.316, *P* = 0.032) and CB2R^–/–^ mice with NCD (PERMANOVA, *R*^2^ = 0.196, *P* = 0.033) were different from those of WT mice with NCD (Fig. 7b). Of note, the microbial structure of CB2R^-/-^ mice was prone to that of NAFLD mice (PERMANOVA, *R*^2^ = 0.242, *P* = 0.095) (Fig. 7b).

The LEfSe analysis identified the discriminative microbes between CB2R^–/–^ mice and WT mice with NCD, WT NAFLD model mice and WT mice with NCD, and CB2R^-/-^ mice with NCD and WT NAFLD model mice (Fig. 7c, Supplementary Fig. S7). Among these statistically different microbial features, we found that Firmicutes as well as *Clostridia* at class level, *Lachnospirale*s, *Oscillospirales* and *Peptostreptococcales Tissierellales* at order level, *Lachnospiraceae*, *Ruminococcaceae*, *Oscillospiraceae*, *Tissierellales* at family level and their genera including *Lachnospiraceae_NK4A136_group*, *Lachnospiraceae_UCG_001*, *Eubacterium_siraeum_group*, *Clostridium_leptum* and *Ruminococcaceae* were obviously decreased in CB2R^–/–^-NCD mice and WT NAFLD model mice when compared to WT-NCD (Fig. 6c). Similarly, Cyanobacteria and its microbes at class, order, family and genus levels including *Vampirivibrionia* and *Gastranaerophilales* were also depleted in CB2R^–/–^-NCD mice and WT NAFLD model mice when compared to WT-NCD mice (Fig. 7c). Besides, gut microbes from Bacteroidota such as *Bacteroidia*, *Bacteroidales*, *Bacteroides_caecimuris* and *Butyricimonas_virosa* and *Eubacterium_xylanophilum_group*, *Eubacterium_nodatum_group* and *UCG_005* of Firmicutes were significantly enriched in WT mice with NCD compared with those with HFD, while showed no significant difference between CB2R^–/–^-NCD mice and WT-HFD mice (Fig. 7c). Meanwhile, Firmicutes as well as its family *Enterococcaceae* and genera including *Lachnospira*, *Tuzzerella*, *Christensenella*, *Faecalibaculum*, *Streptococcus*, *Enterococcus*, and *Christensenella_minuta* and *Streptococcus_danieliae* at species level were decreased in WT mice with NCD while unchanged in CB2R^-/-^ mice with NCD when compared with WT mice with HFD (Fig. 7c). The *Marinifilaceae*, *Odoribacter*, *Rikenella* from Bacteroidota, *Eggerthellaceae* and *Enterorhabdus* of Actinobacteriota, and *Enterobacterales*, *Alcaligenaceae* and *Achromobacter* belonged to Proteobacteria were also depleted in WT-NCD while unchanged in CB2R^-/-^ mice when compared with WT NAFLD model mice (Fig. 7c).

The taxa analysis showed that the phyla including Firmicutes, Desulfobacterota, Actinobacteriota, Patescibacteria, Campilobacterota and Deferribacterota were increased while Bacteroidota, Verrucomicrobiota and Proteobacteria were decreased in HFD-induced NAFLD model mice of WT when compared with WT mice with NCD (Fig. 7d). Of note, the decreased Bacteroidota and increased Actinobacteriota which were shown in both NAFLD patients and mice were also observed in CB2R^-/-^ mice (Fig. 7d). For microbes at family and genus levels, *Marinifilaceae* and its genus *Odoribacter*, *Eubacterium_coprostanoligenes_group*, *Streptococcaceae and its genus Lactococcus*, *Atopobiaceae* and its genus *Coriobacteriaceae_UCG-002*, *Eggerthellaceae* and its genus *Enterorhabdus*, *Rikenella* and *Faecalibaculum* were enriched in WT mice with HFD when compared with WT mice with NCD, while showed no significant difference between WT-HFD mice and CB2R^-/-^-NCD mice (Fig. 7e, f). The enriched Blautia in HFD-induced NAFLD mice was also presented the increased trend in CB2R^–/–^ mice (Fig. 7f). The decreased trend of *Muribaculaceae* and its genus, *Gastranaerophilales* from *Cyanobacteria*, and *Eubacterium_xylanophilum_group* in WT mice with HFD was also shown in CB2R^-/-^ mice (Fig. 7e, f). Since the gut microbiota was known to play a key role in NAFLD development, these findings of the similar alterations in microbial structure of NAFLD model mice and CB2R^-/-^ mice suggested that the NAFLD phenotype in CB2R^-/-^ mice contributed to gut dysbiosis.

Consistent with findings in NAFLD patients, the function prediction results of microbial community showed that HFD-induced NAFLD model mice were featured with function alterations on amino acid metabolism including phenylalanine, tyrosine, tryptophan, alanine, aspartate, glutamate and arginine, vitamin B6 metabolism, fatty acid biosynthesis, citrate cycle, pentose and glucuronate interconversions and oxidative phosphorylation when compared with WT mice with NCD (Fig. 7g). It’s worth noting that among these common functional shifts in NAFLD patients and model mice, the microbes in CB2R^–/–^ mice only exhibited functional alterations on phenylalanine, tyrosine and tryptophan biosynthesis compared to the WT mice (Fig. 7h). Together, these findings supported that the depletion of CB2R might facilitate NAFLD pathogenesis via impacting microbial community especially those involved in phenylalanine, tyrosine and tryptophan biosynthesis.

### Disordered tryptophan metabolism in NAFLD

**Fig. 8:**
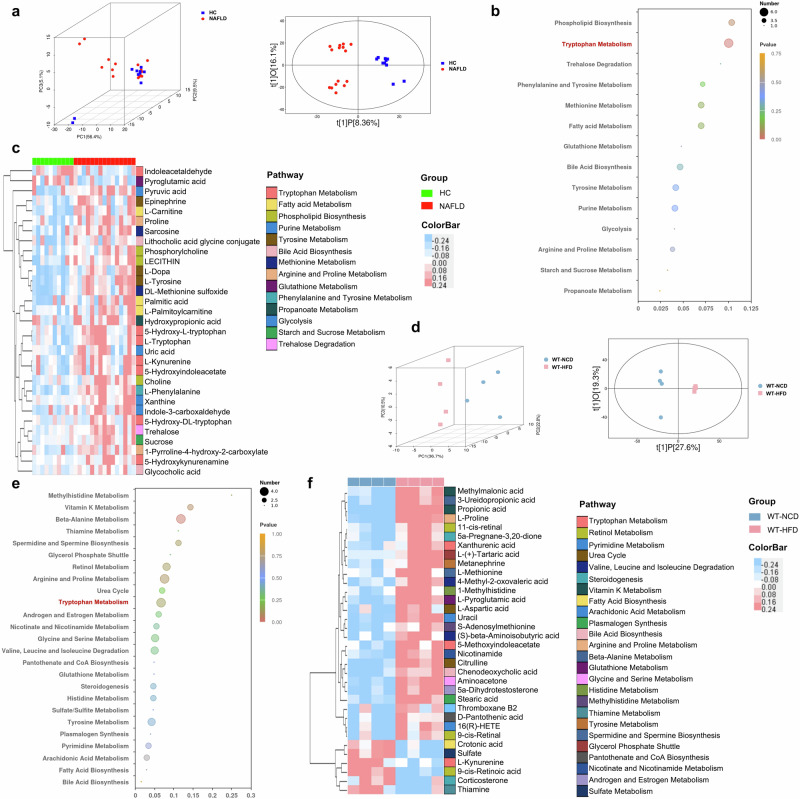
Disordered tryptophan metabolism in NAFLD patients and mice. **a** Three-dimensional score of PCA and two-dimensional score of OPLS-DA model based on the data of serum metabolic profiles of HC and NAFLD patients. **b** The pathway enrichment analysis of differential metabolites between HC and NAFLD patients, (**c**) and the clustering heat map of the differential serum metabolites (The color modules of metabolites correspond to the related pathways). **d** Two and three-dimensional scores of OPLS-DA model based on the data of serum metabolic profiles of WT mice feeding with NCD or HFD. **e** The pathway enrichment analysis of differential metabolites between WT mice with NCD or HFD, (**f**) and the clustering heat map of the differential serum metabolites (The color modules of metabolites correspond to the related pathways). Note: Data were given as median with range. *n*: (**a**–**c**), HC, *n* = 10 and NAFLD, *n* = 15; (**d**–**f**), *n* = 4 per group. PCA principal component analysis, OPLS-DA orthogonal partial least-squares discrimination analysis.

Since metabolites drives the crosstalk between gut microbiota and host, the remarkable functional shift of gut microbiota in metabolism prompts us to perform metabolomics studies. As shown in Fig. 8a, the PCA and OPLS-DA plots showed the differential metabolomics profiles between HC and NAFLD patients. To determine the differential microbial metabolites, the VIP values in OPLS-DA plotting were calculated, and 31 biologically significant metabolites were identified which were mapped to 14 pathways (Fig. 8b**, c**). Metabolites involved in lipid metabolism including fatty acid metabolism (*L*-palmitoylcarnitine, *L*-carnitine, palmitic acid) and phospholipid biosynthesis (phosphorylcholine, choline, lecithin) were increased in NAFLD patients. The amino acid metabolism of arginine and proline (1-pyrroline-4-hydroxy-2-carboxylate, proline) phenylalanine and tyrosine (*L*-phenylalanine, epinephrine, *L*-dopa, *L*-tyrosine) and methionine (sarcosine, *DL*-methionine sulfoxide) was increased while pyroglutamic acid which belongs to glutathione metabolism was decreased in NAFLD patients. The metabolites of bile acid biosynthesis (lithocholic acid glycine conjugate, glycocholic acid), propanoate metabolism (hydroxypropionic acid), purine metabolism (indole-3-carboxaldehyde, uric acid, xanthine), as well as pyruvic acid, sucrose and trehalose were also increased in NAFLD patients compared with HC. Notably, seven of these 31 differential metabolites including *L*-kynurenine, 5-hydroxy-*L*-tryptophan, *L*-tryptophan, 5-hydroxyindoleacetate, 5-hydroxy-*DL*-tryptophan, 5-hydroxykynurenamine and indoleacetaldehyde were annotated to tryptophan metabolism (Fig. 8c). These findings showed that metabolic disturbance especially the altered tryptophan metabolism in NAFLD patients.

The serum metabolic profiles of HFD-induced NAFLD mice were also distinct from WT mice with NCD (Fig. 8d). Consistent with the findings in NAFLD patients, the disturbance of arginine and proline metabolism (*L*-proline), bile acid biosynthesis (chenodeoxycholic acid) and glutathione metabolism (*L*-pyroglutamic acid) were also exhibited in NAFLD mice (Fig. 8e, f). Of note, the remarkably disorders in tryptophan metabolism (xanthurenic acid, 5-methoxyindoleacetate, *L*-kynurenine) were also shown in NAFLD mice (Fig. 8e, f). The NAFLD mice also showed increased metabolites involved in androgen and estrogen metabolism (5α-dihydrotestosterone), nicotinate and nicotinamide metabolism (nicotinamide), plasmalogen synthesis (stearic acid), pyrimidine metabolism (16(*R*)-HETE, uracil), sulfate/sulfite metabolism (sulfate), urea cycle (citrulline, *L*-aspartic acid), vitamin K metabolism (methylmalonic acid), carbohydrate metabolism (pantothenate and CoA biosynthesis) and amino acid metabolism of beta-alanine (3-ureidopropionic acid), glycine and serine (aminoacetone), histidine (1-methylhistidine), methylhistidine (*S*-adenosylmethionine), spermidine and spermine biosynthesis (*L*-methionine), tyrosine (metanephrine), valine, leucine and isoleucine ((*S*)-beta-aminoisobutyric acid, 4-methyl-2-oxovaleric acid) (Fig. 8e, f). The metabolites belong to lipid metabolism including glycerol phosphate shuttle (*L*-(+)-Tartaric acid), fatty acid biosynthesis (crotonic acid), steroidogenesis (5a-Pregnane-3,20-dione, corticosterone), arachidonic acid metabolism (thromboxane B2), retinol metabolism (9-*cis*-retinal, 11-*cis*-retinal, 9-*cis*-retinoic acid), thiamine metabolism (thiamine) and propionic acid were also different in NAFLD mice compared with WT mice with NCD (Fig. 8e, f). Together with the microbial functional prediction results and the metabolomic profiles in both NAFLD patients and mice, we revealed that gut microbiota-mediated disturbance in tryptophan metabolism contributed to NAFLD development.

### CB2R was involved in NAFLD pathogenesis via mediating gut dysbiosis-mediated tryptophan metabolism disorders

**Fig. 9:**
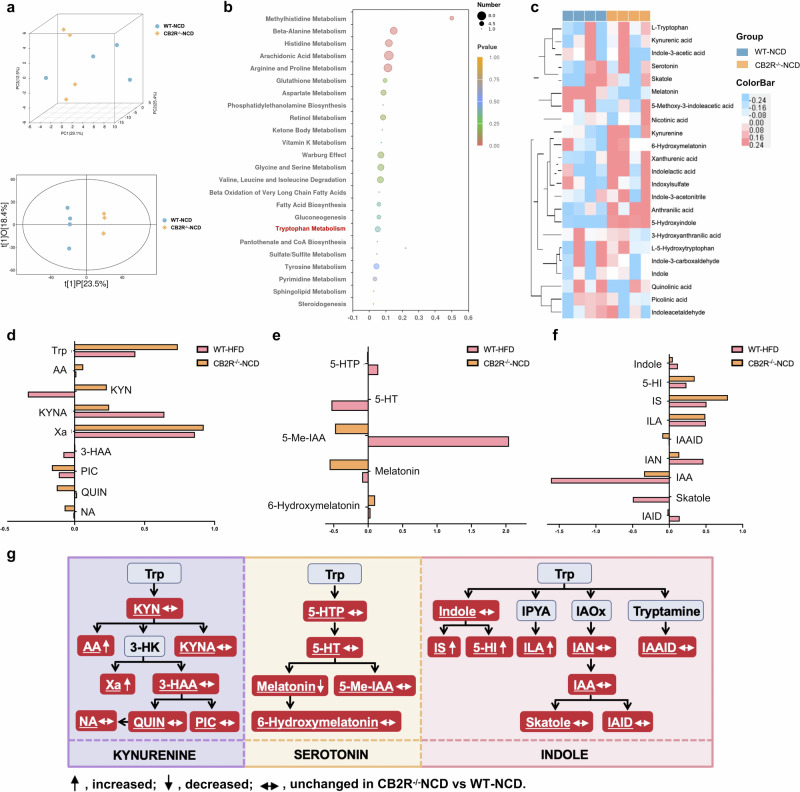
Disordered tryptophan metabolism in CB2R^–/–^ mice. **a** Three-dimensional score of PCA and two-dimensional score of OPLS-DA model based on the data of serum metabolic profiles of WT mice with NCD or CB2R^–/–^ mice with NCD. **b** The pathway enrichment analysis of differential metabolites between WT mice with NCD and CB2R^-/-^ mice with NCD, (**c**) and the clustering heat map of the differential tryptophan metabolites. The tryptophan metabolites belong to (**d**) kynurenine pathway, (**e**) serotonin pathway and (**f**) indole and its derivatives were compared between CB2R^–/–^ mice with NCD and WT mice with HFD. **g** Alterations of tryptophan metabolic pathways in the CB2R^–/–^ mice. The detected tryptophan metabolites in the current study were marked with a white color and underlined. Note: Data were given as medians with range or log2 fold change compared with wild type mice with NCD. *n*: (**a**–**g**), *n* = 4 per group. Trp tryptophan, KYN kynurenine, AA anthranilic acid, 3-HK 3-hydroxykynurenine, KYNA kynurenic acid, Xa xanthurenic acid, 3-HAA 3-hydroxyanthranilic acid, NA nicotinic acid, QUIN quinolinic acid, PIC picolinic acid, 5-HTP *L*-5-hydroxytryptophan, 5-HT serotonin, 5-Me-IAA 5-methoxy-3-indoleacetic, IPYA indole-3-pyruvic acid, IAOx indole-3-acetaldoxime, IS Indoxylsulfate, 5-HI 5-Hydroxyindole, ILA indolelactic acid, IAN indole-3-acetonitrile, IAAID indoleacetaldehyde, IAA indole-3-acetic acid, IAID indole-3-carboxaldehyde.

To explore the metabolites under the regulation of CB2R, we compared the metabolomics profiles of CB2R^–/–^ mice and WT mice with NCD. As shown in Fig. 9a, both the PCA and OPLS-DA plots exhibiting the metabolomics profiles of CB2R^–/–^ mice were distinct from WT mice with NCD. Consistent with the findings of NAFLD patients and mice, the differential metabolites were also enriched in arginine and proline metabolism and glutathione metabolism (Fig. 9b). It was noteworthy that the obviously disordered tryptophan metabolism in both NAFLD patients and mice were also exhibited in CB2R^–/–^ mice (Fig. 9b). As presented in Fig. 9c, a total of 23 tryptophan metabolites were detected in our study. Among which, nine metabolites were annotated to kynurenine pathway, five to serotonin pathway and nine to indole and indole derivatives (Fig. 9d–f). And 19 in 24 metabolites showed the same alteration trend in NAFLD mice and CB2R^–/–^ mice when compared with WT mice with NCD (Fig. 9d). Among these 19 metabolites, xanthurenic acid (Xa) in kynurenine pathway was significantly increased while melatonin which belongs to serotonin pathway was decreased in CB2R^–/–^ mice (Fig. 9d, e, g). Interestingly, indoxylsulfate, 5-hydroxyindole, idolelactic acid (ILA) involved in indole and indole derivatives metabolism which was the major tryptophan metabolism in the intestine under the control of gut microbiota were significantly increased in CB2R^–/–^ mice compared with WT mice with NCD (Fig. 9f, g). These findings suggested that the depletion of CB2R might promote NAFLD progression via disturbing gut microbiota-mediated tryptophan metabolism.

**Fig. 10:**
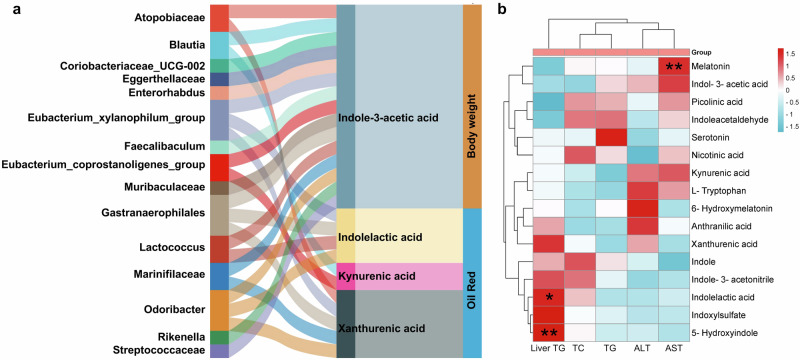
CB2R-mediated gut dysbiosis contributed to NAFLD development in association with tryptophan metabolites. **a** Correlation network showed that gut dysbiosis in CB2R^–/–^ mice was related to dysregulation of tryptophan metabolites, which were associated with parameters reflecting the severity of NAFLD including oil red positive area and body weight. **b** Correlation network between tryptophan metabolites and serum liver marker in CB2R^–/–^ mice. *n*: (**a**, **b**), *n* = 4 per group.

Furthermore, the correlation analysis was performed to reveal the interplay between gut dysbiosis and the perturbed tryptophan metabolism under the regulation of CB2R. As shown in Fig. 10a, microbes including *Atopobiaceae*, *Blautia*, *Coriobacteriaceae_UCG002*, *Eggerthellacea*e, *Enterorhabdus*, *Faecalibaculum*, *Lactococcus*, *Marinifilaceae*, *Odoribacter*, *Rikenella* and *Streptococcaceae* were positively while *Eubacteriumcoprostanoli genes group*, *Muribaculaceae* and *Gastranaerophilales* were negatively associated with indole-3-acetic acid or kynurenic acid. The enriched *Atopobiaceae*, *Marinifilaceae* and *Odoribacter* and depleted *Eubacteriumxylanophilum group* and *Gastranaerophilales* were related to high levels of Xa in CB2R^–/–^ mice. And the *Eubacteriumxylanophilum group*, *Gastranaerophilales*, *Lactococcus* and *Odoribacter* were also associated with increased ILA of CB2R^-/-^ mice. Notably, the indole-3-acetic acid which showed decreased trend in CB2R^–/–^ mice was negatively associated with body weight, while the significantly enriched ILA and Xa as well as kynurenic acid which showed increased trend in CB2R^–/–^ mice were positively associated with the oil red positive area in the liver. In addition, the significantly decreased melatonin in CB2R^–/–^ mice was positively associated with serum levels of AST, and the significantly enriched ILA and 5-hydroxyindole were positively associated with hepatic TG content (Fig. 10b). These findings implied that the perturbed tryptophan metabolism which was mediated by gut dysbiosis was related to the development of NAFLD in CB2R^–/–^ mice.

### Tryptophan metabolite ILA enriched in CB2R^–/–^ mice aggravated hepatic steatosis

**Fig. 11:**
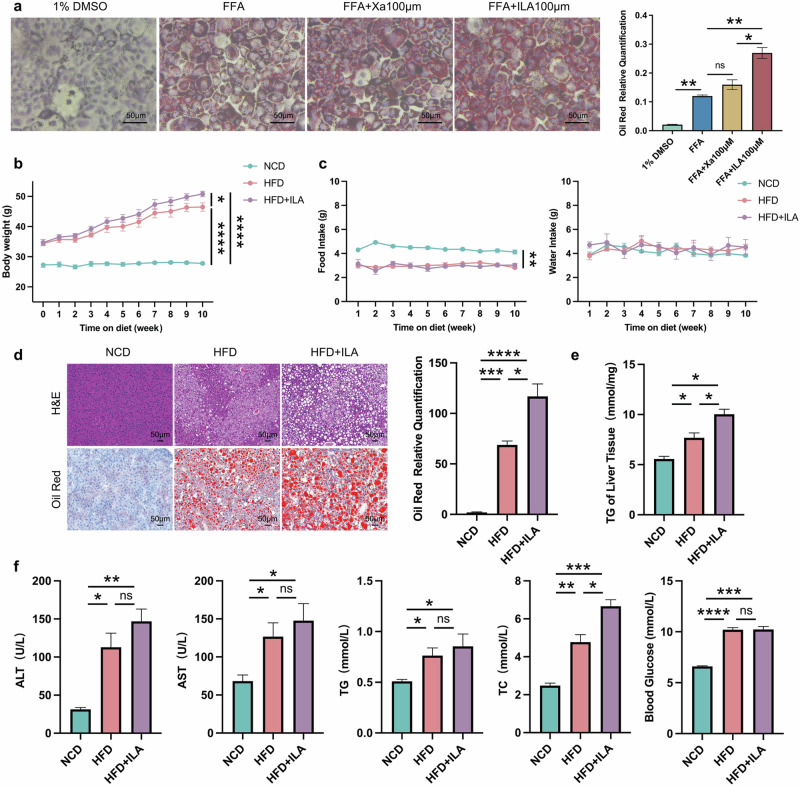
The disordered tryptophan metabolites indole-3-lactic acid aggravates lipid deposition. **a** AML12 hepatocytes were pre-treated with 1% DMSO, Xa or ILA and then exposed or not to FFA. The representative images (400×) of Oil red O staining and the morphometric analysis for quantification. **b** The body weight of WT mice with NCD and WT NAFLD model mice treated or not with ILA throughout the experimental period was recorded. **c** The food intake and water intake. **d** The representative images (200×) of H&E or Oil red O staining, and the morphometric analysis of Oil red O staining for quantification. **e** The comparison of liver homogenete levels of TG. **f** The comparison of serum levels of transaminases (ALT, AST) and lipid parameters (TG, TC), and blood glucose between WT mice with NCD and WT NAFLD model mice treated or not with ILA. Note: Data were given as mean ± SEM. *n*: (**a**), *n* = 3 per group; (**b**), NCD and HFD + ILA, *n* = 4 and HFD, *n* = 3; (**c**–**f**), *n* = 3 per group. *P < 0.05; **P < 0.01; ***P < 0.001; ****P < 0.0001; ns no significance. FFA free fatty acid (a mixture of oleic acid and palmitic acid), DMSO dimethyl sulfoxide.

To validate the effect of enriched tryptophan metabolites of CB2R^–/–^ mice in NAFLD, we performed both in vitro and in vivo experiments. Lipid droplet accumulation was increased significantly within AML12 hepatocytes when exposed to ILA compared to free fatty acid (FFA) induced alone (Fig. 11a), while Xa-treated cells showed a slight, non-statistically significant increase of lipid accumulation compared model group (Fig. 11a). Meanwhile, as shown in Fig. 11b, c, the body weight of ILA-treated mice fed with HFD was higher than NAFLD model mice, and average food intake and water intake has no difference. Moreover, histopathology of liver sections demonstrated that ILA-treated mice fed with HFD had significantly more steatosis in comparison to model mice (Fig. 11d). Accordingly, the levels of serum TC and hepatic TG content were significantly increased in ILA-treated fed with HFD mice compared to model mice (Fig. 11e, f). These results indicated that enriched tryptophan ILA in CB2R^-/-^ mice significantly aggravates NAFLD phenotypes, and further verified the impact of CB2R on regulation of microbiota in NAFLD was associated with tryptophan metabolism.

## Discussion

In the current study, we found that NAFLD model mice were presented with the depletion of CB2R and CB2R^–/–^ mice with NCD developed NAFLD. Interestingly, microbiota transplantation from WT mice ameliorated the NAFLD phenotype of CB2R^–/–^ mice. Further, we revealed that the microbial structure including the increased Actinobacteriota and decreased Bacteroidota of CB2R^–/–^ mice was resemble to that of NAFLD patients and model mice. Notably, the microbial functional analysis and metabolomics profiling results together pointed out obviously disturbed tryptophan metabolism in NAFLD patients and mice, which were also exhibited in CB2R^-/-^ mice. Correlation network presented that the disordered tryptophan metabolites such as ILA and Xa were modulated by gut dysbiosis and related to NAFLD severity indicators of obesity, oil red positive area, serum AST and liver TG content. Treatment with enriched tryptophan metabolites, especially ILA, aggravated hepatic steatosis in the in vitro and in vivo model of NAFLD. These results demonstrated the crucial role of CB2R in the pathogenesis of NAFLD. And the underlying mechanisms were linked to gut microbiota-mediated perturbed tryptophan metabolism. Our novel findings suggested CB2R and the associated microbes and tryptophan metabolites as promising targets for NAFLD.

The present study showed that CB2R expression was obviously decreased in NAFLD mice. And CB2R^–/–^ mice developed NAFLD even feeding with NCD. CB2R was widely recognized in the modulation of inflammatory process, while it has been acting as an underappreciated aspect of eCB signaling in metabolic disorders. The role of the endocannabinoid system in metabolic diseases was long-term known mainly attributed to the CB1R. For example, it was reported that the heightened eCB/CB1R activity was related to the susceptibility of metabolic diseases among racial groups. Studies demonstrated that the CB1R overactivity contributed to hepatic steatosis. Therefore, CB1Rs was considered as a promising clinical target for the treatment of metabolic disorders especially NAFLD which lacking FDA-approved drugs. However, the adverse psychiatric effects of CB1R antagonists discontinued the developing of CB1R blockers. Functional studies indicated opposing effects of CB2R and CB1R in liver fibrosis, whereas the role of CB2R in hepatic steatosis was unknown. In this study, we revealed that mice lacking CB2R developed NAFLD, supporting that the depletion of CB2R promoted NAFLD progression. These results recommended that selective modulation of CB2R signaling may provide novel insights for the treatment of NAFLD.

Here, we uncovered the microbial community of CB2R^–/–^ mice including the high abundance of Actinobacteriota and *Blautia*, and the low abundance of Bacteroidota and *Muribaculaceae* was similar to the trend of NAFLD patients and mice. Plenty of studies demonstrated the causative role of the dysbiotic gut microbiota in the development of NAFLD. In line with our results, the increased Actinobacteriota and decreased Bacteroidota were also reported in NAFLD models and humans [36]. And the depleted *Muribaculaceae* and *Eubacterium xylanophilum* levels observed in our CB2R^-/-^ mice were shown to be negatively correlated to HFD-induced biochemical and pathological indexes [36, 37]. The supplementation of *Blautia* caused liver inflammation and fibrosis and promoted the progression of NAFLD in mice [38]. Whereases, the underlying mechanisms by which NAFLD induced gut dysbiosis has not yet been figured out. The phenomenon that CB2R^-/-^ mice housed with WT mice ameliorated NAFLD phenotypes in our study indicated that CB2R mediated the NAFLD-associated microbial signatures. Actually, the modulating role of eCB signaling in gut microbiota has been reported in inflammatory diseases such as ARDS, colitis, autoimmune encephalomyelitis and HIV/SIV infection [39, 40]. Interestingly, the microbial similarity of CB2R^–/–^ mice and NAFLD model mice seems to prevent FMT colonization [41, 42], and CB2R^–/–^ mice transplanted microbiota from NAFLD mice did not aggravate NAFLD phenotype (Fig. 4a–e). Accordingly, our novel findings revealed that one of the mechanisms by which CB2R contributed to NAFLD development was through regulating gut microbiota.

The microbial functional analysis noted that CB2R modulated the microbial activity in tryptophan metabolism, which was supported by metabolomics findings. The endocannabinoid signaling system was considered as an important regulator of energy homeostasis. The mechanisms by which endocannabinoid signaling modulates metabolism especially lipid metabolism have been partially investigated. On the one hand, endocannabinoids are bioactive fatty acid amides and esters in nature [43]. For instance, 2-arachidonoylglycerol which is the most abundant endocannabinoid, was known as an endogenous lipid signaling mediator and its immediate metabolite arachidonic acid was an essential fatty acid [44]. On the other hand, anandamide, another endocannabinoid, was reported to increase adipocyte differentiation partially through inducing PPAR-γ gene expression [43]. Since both 2-arachidonoylglycerol and anandamide were effective ligands of CB2R, the above-mentioned mechanisms failed to explain the reason why depletion of CB2R promoted NAFLD development. Considering the relevance of eCB system and microbial metabolites in the manipulation of metabolic process, our findings indicated the tryptophan metabolites might be the critical effector of CB2R in facilitating NAFLD development.

Furthermore, the correlation network linked the significantly enriched tryptophan metabolites including ILA and Xa to NAFLD severity parameters of obesity and oil red positive area in CB2R^-/-^ mice. And these perturbed metabolites were associated with the increased Actinobacteriota genera and decreased *Eubacterium xylanophilum*. A large array of metabolites drives the interaction between gut microbiota and the host. Among which, the tryptophan metabolites were the most studied categories. Tryptophan was directly converted into indole and its derivatives such as ILA, which were directly controlled by intestinal microorganisms. Previous in vitro experiment revealed that Actinobacteriota genus produced ILA [45]. Here, we noticed the linkage between disordered Actinobacteriota genera and enriched ILA in CB2R^-/-^ mice. As one of the indole derivatives, ILA was considered to be almost harmless in previous studies of inflammatory diseases [46]. Similar to ILA, Xa modulated by gut microbiota was another AhR ligand [47]. These typtophan metabolites could maintain immune homeostasis of the intestine, skin and tumor microenvironment in an AhR-dependent manner [48–50]. However, patients and mice with metabolic syndromes including NAFLD were characterized by increased ILA and Xa as reported in our or previous studies [51–53]. Recent researches in metabolic diseases showed that enhancing AhR expression promoted obesity and insulin resistance. And activating hepatic AhR signaling led to liver steatosis [54–56]. Based on these findings, we speculated that the microbial mediated-ILA and Xa enrichment might promote NAFLD development in CB2R^-/-^ mice via activating AhR.

There were still several limitations in our study. Firstly, this study mainly focused on the role of CB2R in the metabolic disorders of NAFLD, whether CB2R depletion affect the inflammatory process especially in the progressive NASH stage required further investigation. Secondly, the concrete mechanism by which CB2R modulated gut microbiota deserved systematic researches. And the essential role of tryptophan metabolites in CB2R-mediated NAFLD development should be validated. Lastly, it is worth exploring the putative influence of CB2R upon CB1R signaling during NAFLD progression.

In conclusion, a major finding of our study was that the depletion of CB2R contributed to NAFLD progression via modulating gut microbiome. We suggested tryptophan metabolites as microbial messengers to be involved in CB2R-mediated NAFLD development. These novel findings highlighted the role of CB2R in metabolic process and revealed the modulating effect CB2R in microbial community of NAFLD. This CB2R-microbiome-tryptophan metabolites axis might open a new avenue for cannabinoid system-based therapeutics to prevent and treat NAFLD. However, more clinical investigations are essential to validate this concept.

## Supplementary information

Supplementary Material and Method

Supplementary Fig. S1

Supplementary Fig. S2

Supplementary Fig. S3

Supplementary Fig. S4

Supplementary Fig. S5

Supplementary Fig. S6

Supplementary Fig. S7

Supplementary Table 1

## Data Availability

The sequence dataset of the fecal microbiota can be downloaded from the National Center for Biotechnology Information (NCBI) Bioproject database (PRJNA1064705).
