# Supplementary material for: Cannabinoid-2 receptor depletion promotes non-alcoholic fatty liver disease in mice via disturbing gut microbiota and tryptophan metabolism
Source: Acta Pharmacol Sin. 2025 Feb 20;46(6):1676–91. doi: 10.1038/s41401-025-01495-w (PMC12098919; doi:10.1038/s41401-025-01495-w)
Supplement: Supplementary file 1 — Supplementary Material and Method [file 41401_2025_1495_MOESM1_ESM.docx]

1. **Material and Method**

1.1. Fecal Bacteria Quantification

Commercial animal stool DNA extraction kit (EK1212, ECOTOP, Guangzhou, China) were used for fecal bacteria quantification as recommended by the manufacturers. Briefly, fecal samples were weighted, resuspended in Inhibit EX buffer and homogenized for 1 min. The suspensions were incubated at 85 ℃ for 2 min, vortexed for 15 s, centrifuged at 14000 g for 2 min and keep 200 μL supernatant. After 15 μL proteinase K and 200 μL buffer AL were added, the mixtures were incubated at 70 °C for 10 min. Then 500 μL of anhydrous ethanol was added, and the mixtures were applied to spin columns. After centrifugation, the spin columns were added with 500 μL buffer AW1 or AW 2 and centrifuged for 1 min. Buffer ATE (5uL per 10 mg fecal sample) was directly pipetted onto the spin columns membrane and then centrifuged for 2 min to elute DNA. The Total bacterial DNA concentration was detected by qRT-PCR using forward primer (5’-GTGSTGCAYGGYYGTCGTCA-3’) and reverse primer (5’-ACGTCRTCCMCN CCTTCCTC-3’). Subsequently, genomic DNA copies were determined via a standard curve.

1.3. Immunofluorescence Staining

The liver paraffin sections were harvested as described above, and then deparaffinized, rehydrated, antigen retrieval, serum blocking, primary antibody incubated (CB2R, ab3559, Abcam, Cambridge, MA, USA; CK18, Servicebio, Hubei, China), secondary antibody incubated and DAPI stained. All images were acquired using Axio Scope A1 (Zeiss, Oberkochen, Germany).

1.4. Quantitative Real-time PCR Analysis

Total RNA was extracted using RNAiso Plus kit (Takara, Tokyo, Japan) as suggested by the manufacturer. The complementary DNA (cDNA) was synthesized using PrimeScript^TM^RT reagent kit (Takara, Tokyo, Japan), and expression levels of genes were measured by qRT-PCR using hamQ Universal SYBR qPCR Master Mix (Vazyme, Jiangsu, China) in CFX Maestro system (BioRad, Hercules, CA, USA). GAPDH mRNA was used as the control gene. The primer sequences were as follows: CNR2 forward, 5’-ACGGTGGCTTGGAGTTCAAC-3’; CNR2 reverse, 5’-GCCGGGAGGACAGGATAAT-3’; GAPDH forward, 5’-AGGTCGGTGTGAACGGATTTG-3’; and GAPDH reverse, 5’-TGTAGACCATG-TAGTTGAGGTCA-3’. Gene expression levels were calculated based on the ΔΔCT relative qualification method.

**Figures legends**

**Supplementary Figure s1. The phenotype of WT mice with NCD, WT NAFLD model mice and CB2R^-/-^ mice with NCD.** **a** The body weight of WT mice with NCD, WT NAFLD model mice or CB2R^-/-^ mice with NCD throughout the experimental period were recorded. **b** The food intake and water intake. **c** The Representative images (200×) of H&E or Oil red O staining, and the morphometric analysis of Oil red O staining for quantification. **d** The comparison of liver homogenate levels of TG. **e** The comparison of serum levels of transaminases (ALT, AST) and lipid parameters (TG, TC), and blood glucose between WT mice with NCD, WT NAFLD model mice or CB2R^-/-^ mice with NCD. Note: Data were given as mean ± SEM. *n*: a-b, WT-NCD and CB2R^-/-^-NCD, *n* = 4 and WT-HFD, *n* = 3; c-e, *n* = 3 per group. **P* < 0.05; ***P* < 0.01; ****P* < 0.001; *****P* < 0.0001; ns, no significance. Abbreviations: NCD, normal control diet; HFD, high fat diet; WT, wild type; CB2R, cannabinoid-2 receptor; H&E, hematoxylin & eosin; ALT, alanine transaminase; AST, aspartate transferase; TC, total cholesterol; TG, triglyceride; SEM, standard error of mean.

**Supplementary Figure s2. The expression of CB2R in different organs between WT mice with NCD, WT NAFLD model mice or CB2R^-/-^ mice with NCD.** **a** Quantitative real-time PCR analysis of CB2R in liver, intestine, spleen, lymph nodes, pancreas, adipose, muscle and bone between WT mice with NCD, WT NAFLD model mice and CB2R^-/-^ mice with NCD. **b** Immunohistochemically labeled with CB2R of mice liver sections and quantitative analysis (200×, 400×) between WT mice fed with NCD or HFD. **c** Immunohistochemically labeled with CB2R of mice intestine sections and quantitative analysis (200×, 400×) between WT mice with NCD or HFD. **d** Immunofluorescence staining of CB2R (green) and CK18 (hepatocytes, red) in liver of WT mice. Nuclei are stained with DAPI (blue). Note: Data were given as mean ± SEM. *n*: a-d, *n* = 3 per group. **P* < 0.05; ***P* < 0.01; ****P* < 0.001; *****P* < 0.0001; ns, no significance. Abbreviations: CK18, cytokeratin18.

**Supplementary Figure s3. Genotyping Assay for Mouse Tail.** WT primers including forward primer (5’-GGAGTTCAACCCCATGAAGGAGTAC-3’) and reverse primer (5’-GACTAGAGCTTTGTAGGTAGGCGG-G-3’), CB2R knock out primers including forward primer (5’-GGGGATCGATCCGTCCTGTAAGTCT-3’) and reverse primer (5’-GACTAGAGCTTTGTAGGTAGGCGGG-3’).

**Supplementary Figure s4. RNA-sequencing analysis of liver transcriptome between CB2R^-/-^ mice and WT NCD mice.** **a** The volcano plots of DEGs in CB2R^-/-^ mice compared to WT mice (blue, down-regulated; red, up-regulated). **b** The clustering heat map of DEGs. **c** The GO and **d** KEGG pathway enrichment analysis of DEGs between CB2R^-/-^ mice and WT mice. **e** GSEA of fatty acid biosynthetic process, **f** fatty acid oxidation and **g** fatty acid transport in CB2R^-/-^ mice compared to WT mice. **h** Gene sets of lipid metabolism. *n*: a-h, *n* = 4 per group. Abbreviations: DEGs, differential expressed genes; GSEA, gene set enrichment analyse.

**Supplementary Figure s5. The severity of NAFLD in adult CB2R^-/-^ mice were improved when cohousing with WT mice.** **a** The body weight, food intake of 6-week-old CB2R^-/-^ mice cohousing with WT mice or CB2R^-/-^ mice throughout the experimental period were recorded. **b** The representative images (200×) of H&E or Oil red O staining, and the morphometric analysis of Oil red O staining for quantification. **c** The comparison of serum levels of transaminases (ALT, AST) and lipid parameters (TG, TC), and blood glucose between CB2R^-/-^ mice cohousing with WT mice or CB2R^-/-^ mice. **d** The comparison of liver homogenate levels of TG. Note: Data were given as mean ± SEM. *n*: a-d, *n* = 5 per group. **P* < 0.05; ***P* < 0.01; ns, no significance. Abbreviations: Co, cohousing.

**Supplementary Figure s6. The phenotype of WT mice cohoused with CB2R^-/-^ mice.** **a** The body weight, food intake of 6-week-old WT mice cohousing with CB2R^-/-^ mice or WT mice throughout the experimental period were recorded. **b** The representative images (200×) of H&E or Oil red O staining, and the morphometric analysis of Oil red O staining for quantification. **c** The comparison of serum levels of transaminases (ALT, AST) and lipid parameters (TG, TC), and blood glucose between WT mice cohousing with CB2R^-/-^ mice or WT mice. **d** The comparison of liver homogenate levels of TG. Note: Data were given as mean ± SEM. *n*: a-d, Co-WT, *n* = 3 and Co-CB2R^-/-^, *n* = 5. **P* < 0.05; ***P* < 0.01; ns, no significance.

**Supplementary Figure s7. LEfSe analysis between WT NAFLD model mice and WT mice with NCD, and CB2R^-/-^ mice with NCD and WT NAFLD model mice.** Abbreviations: NAFLD, nonalcoholic fatty liver disease.

**Table legends**

**Supplementary Table s1. Baseline characteristics of patients.** Abbreviations: HC, healthy control, NAFLD, nonalcoholic fatty liver disease, BMI, body mass index; ALT, alanine transaminase; AST, aspartate transferase; LDL, low-density lipoprotein; HDL, high-density lipoprotein; SEM, standard error of mean.
