# Supplementary figures and images for: Cannabinoid-2 receptor depletion promotes non-alcoholic fatty liver disease in mice via disturbing gut microbiota and tryptophan metabolism
Source: Acta Pharmacol Sin. 2025 Feb 20;46(6):1676–91. doi: 10.1038/s41401-025-01495-w (PMC12098919; doi:10.1038/s41401-025-01495-w)

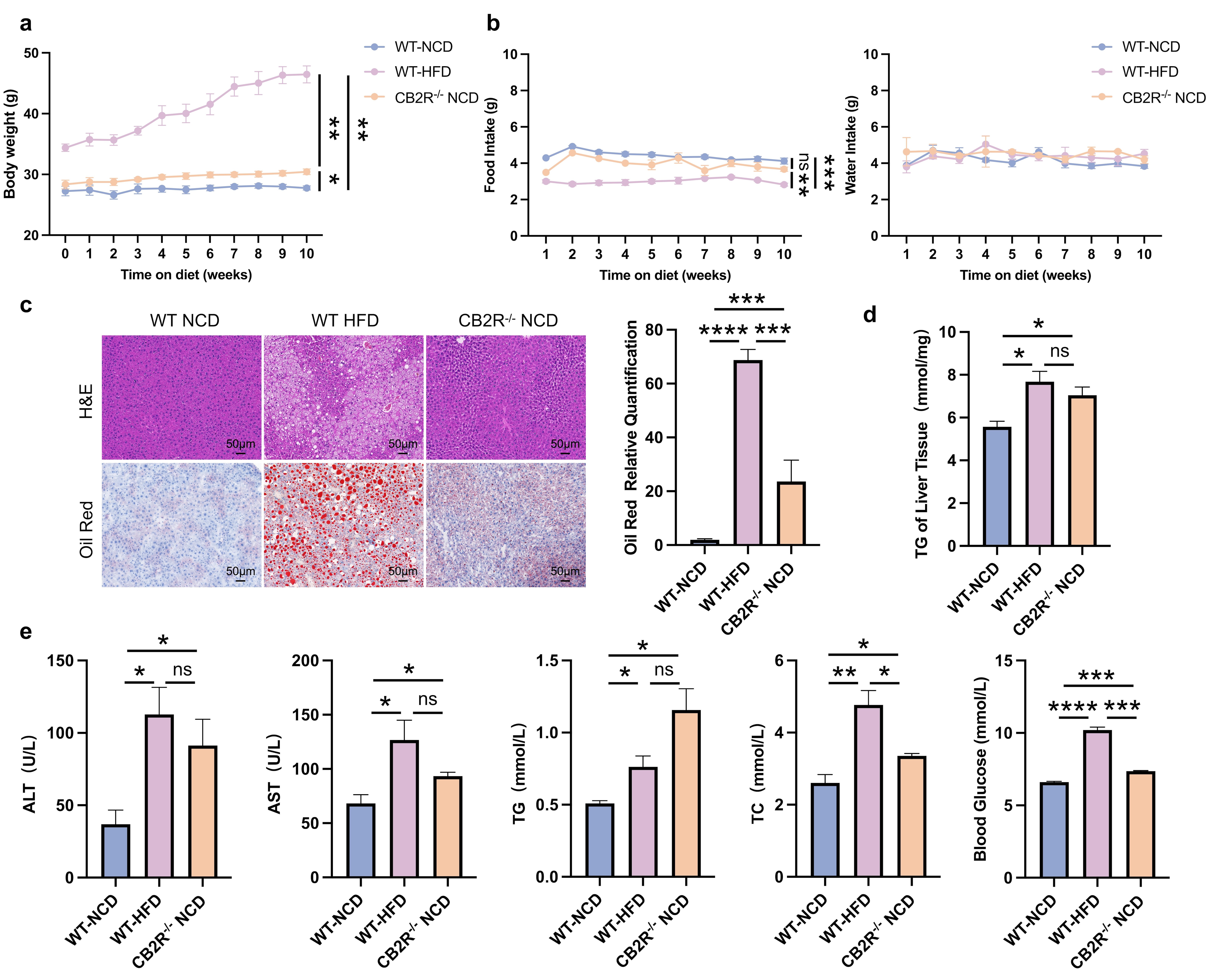

Supplement: Supplementary file 2 — Supplementary Fig. S1 [file 41401_2025_1495_MOESM2_ESM.jpg]

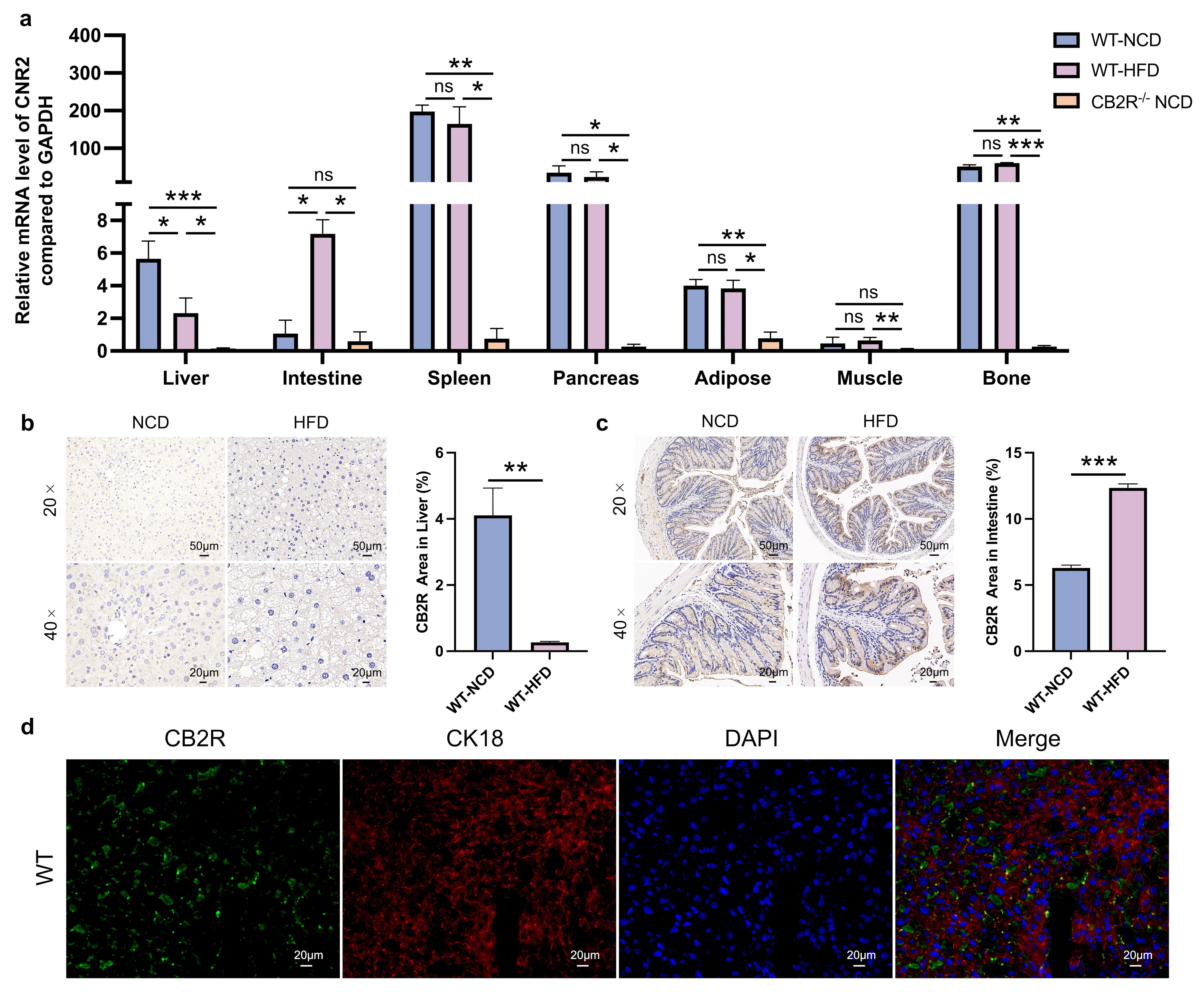

Supplement: Supplementary file 3 — Supplementary Fig. S2 [file 41401_2025_1495_MOESM3_ESM.jpg]

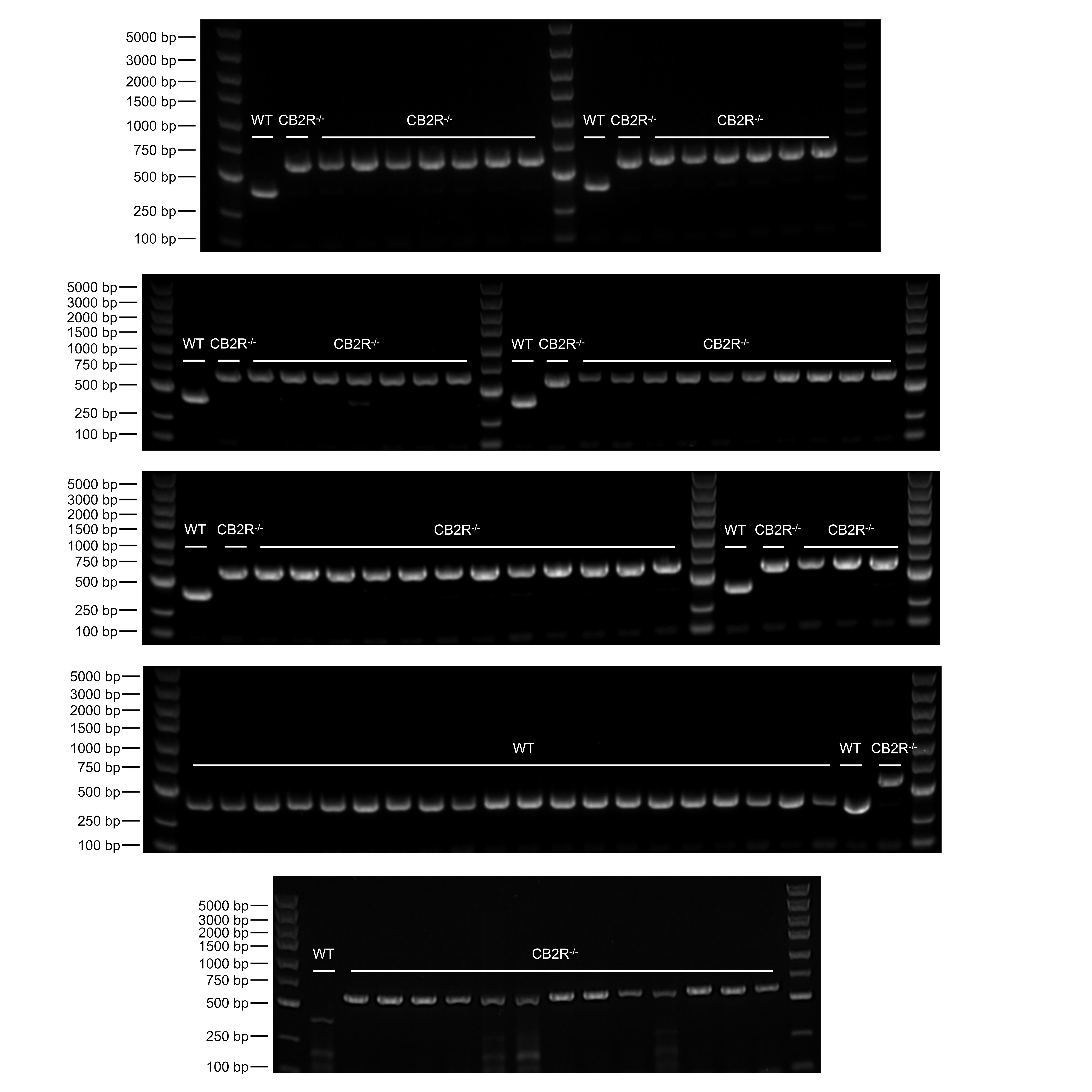

Supplement: Supplementary file 4 — Supplementary Fig. S3 [file 41401_2025_1495_MOESM4_ESM.jpg]

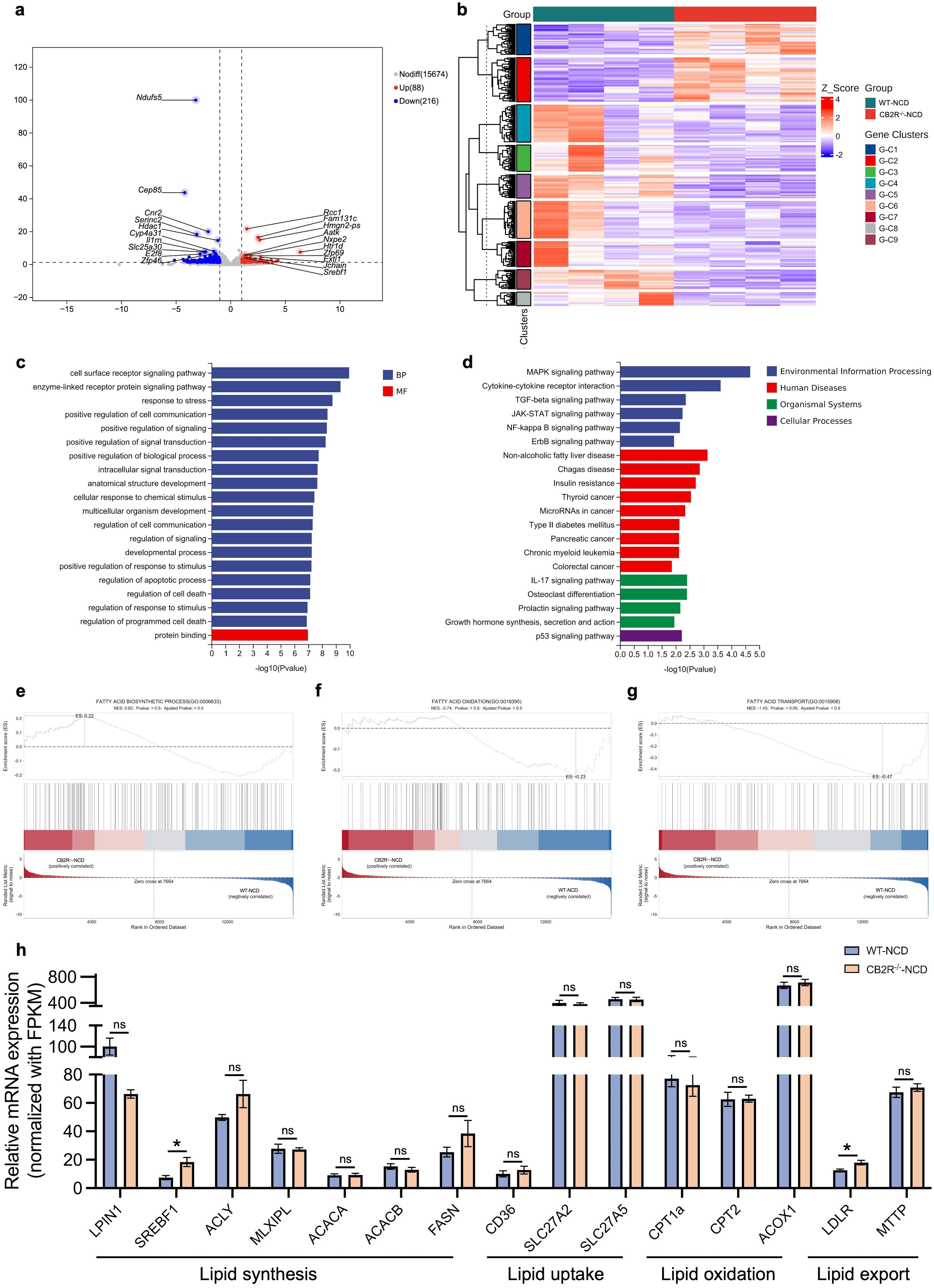

Supplement: Supplementary file 5 — Supplementary Fig. S4 [file 41401_2025_1495_MOESM5_ESM.jpg]

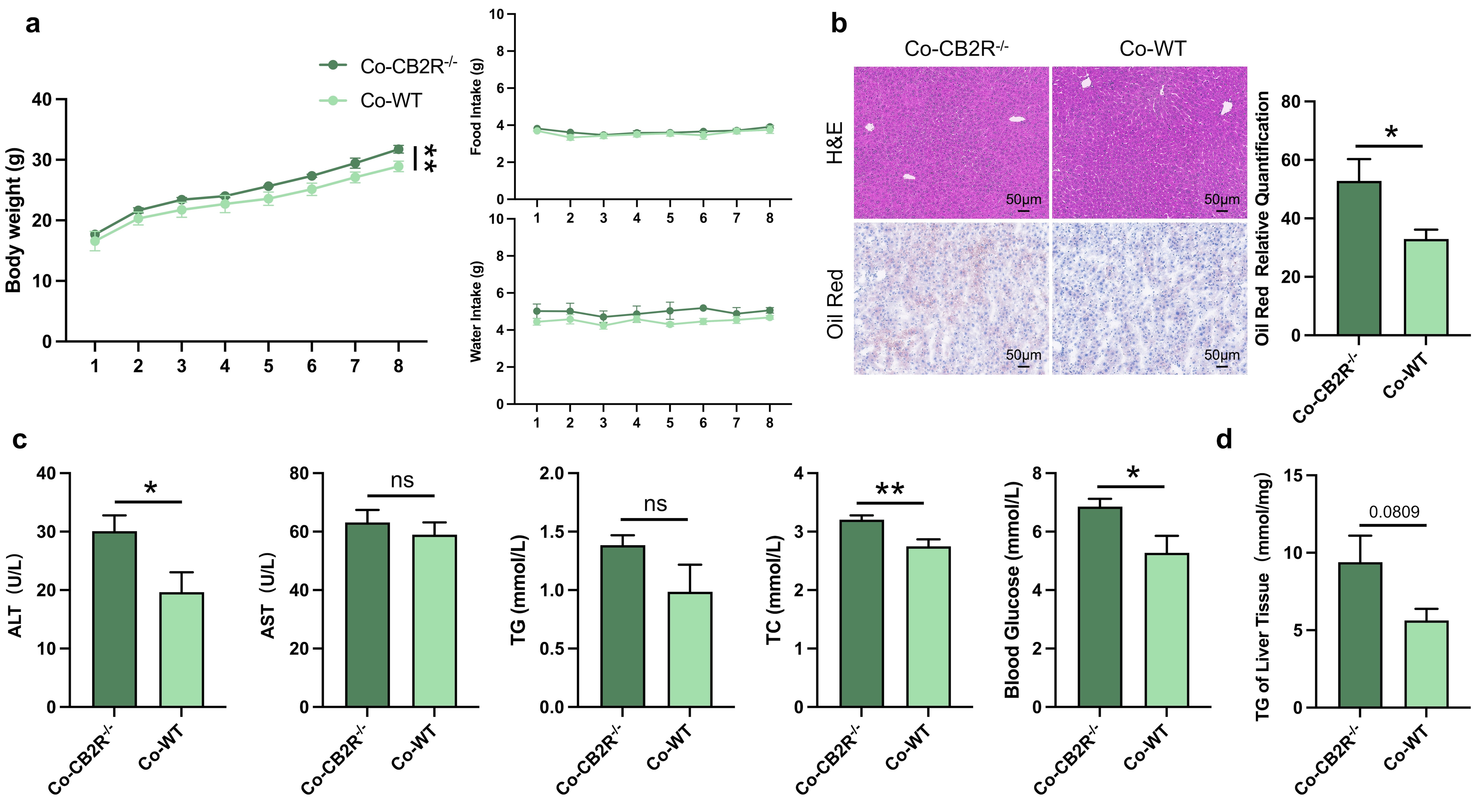

Supplement: Supplementary file 6 — Supplementary Fig. S5 [file 41401_2025_1495_MOESM6_ESM.jpg]

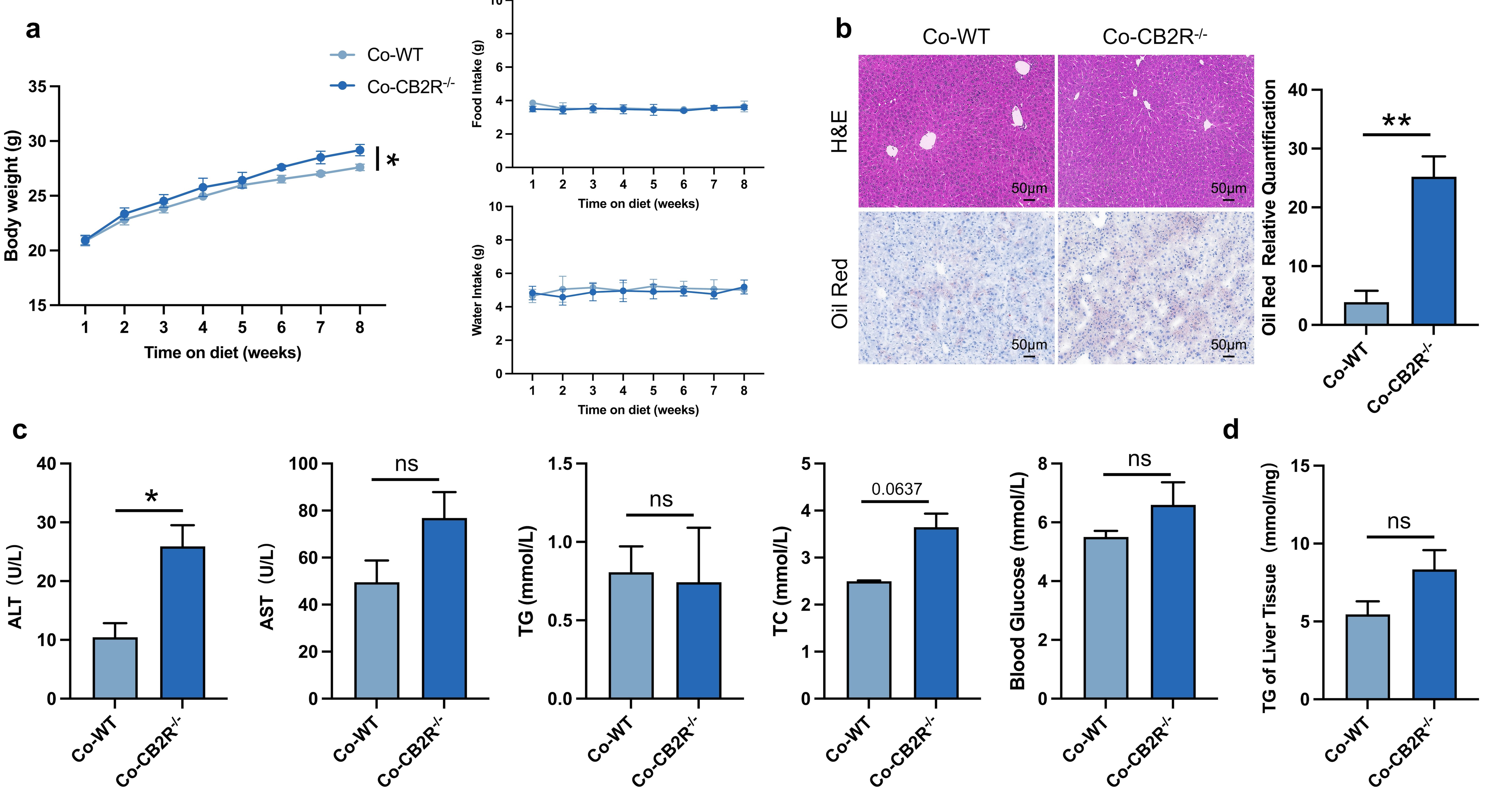

Supplement: Supplementary file 7 — Supplementary Fig. S6 [file 41401_2025_1495_MOESM7_ESM.jpg]

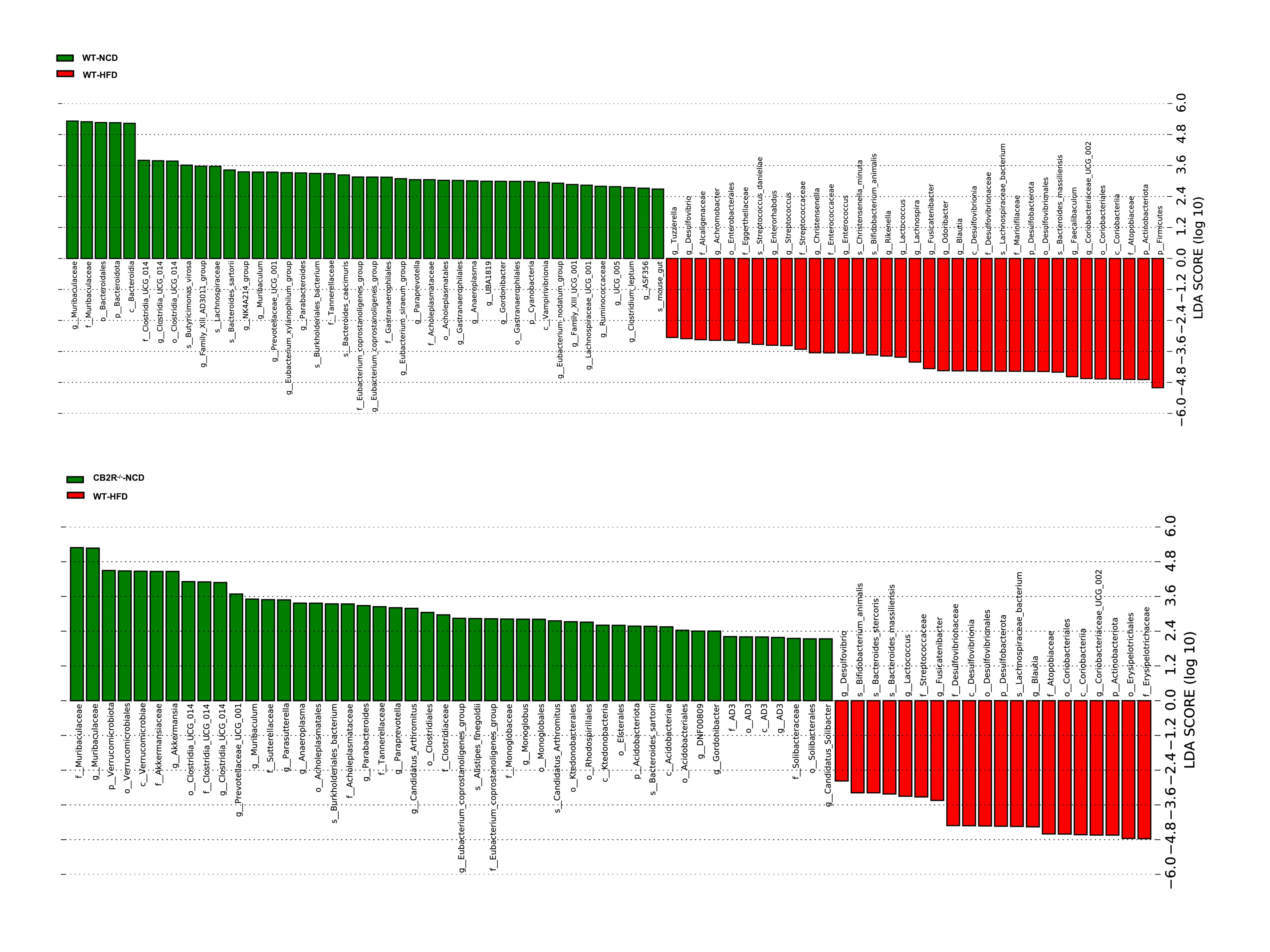

Supplement: Supplementary file 8 — Supplementary Fig. S7 [file 41401_2025_1495_MOESM8_ESM.jpg]
