# Supplementary material for: Cannabinoid-2 receptor depletion promotes non-alcoholic fatty liver disease in mice via disturbing gut microbiota and tryptophan metabolism
Source: Acta Pharmacol Sin. 2025 Feb 20;46(6):1676–91. doi: 10.1038/s41401-025-01495-w (PMC12098919; doi:10.1038/s41401-025-01495-w)
Supplement: Supplementary file 9 — Supplementary Table 1 [file 41401_2025_1495_MOESM9_ESM.docx]

| **Table 1. Demographic and clinical parameters of HC and NAFLD groups.** | | | |
| --- | --- | --- | --- |
|  | HC | NAFLD | *p* value |
| n | 10 | 15 |  |
| Age (years) | 38 ± 3 | 42 ± 4 | 0.425 |
| **Sex** |  |  | 0.072 |
| Female | 5 (50%) | 2 (13%) |  |
| Male | 5 (50%) | 13 (87%) |  |
| **Body composition** |  |  |  |
| Body weight (kg) | 59 ± 3 | 74 ± 4 | 0.009** |
| Height (cm) | 166 ± 2 | 165 ± 2 | 0.55 |
| BMI (kg/m^2^) | 21.3 ± 0.9 | 26.8 ± 0.5 | <0.0001**** |
| **Liver enzymes** |  |  |  |
| ALT (U/L) | 19.0 ± 2.5 | 50.6 ± 8.8 | 0.026* |
| AST (U/L) | 22.0 ± 0.8 | 33.5 ± 4.1 | 0.076 |
| **Plasma lipids** |  |  |  |
| Triglycerides (mmol/L) | 0.98 ± 0.12 | 2.65 ± 0.34 | 0.004** |
| LDL cholesterol (mmol/L) | 3.14 ± 0.3 | 3.26 ± 0.19 | 0.705 |
| HDL cholesterol (mmol/L) | 1.53 ± 0.12 | 1.14 ± 0.04 | 0.001** |
| Total cholesterol (mmol/L) | 4.88 ± 0.13 | 4.76 ± 0.35 | 0.833 |
| **Others** |  |  |  |
| Blood glucose (mmol/L) | 4.6 ± 0.1 | 5.2 ± 0.2 | 0.092 |
| Uric acid (μmol/L) | 317.8 ± 21.0 | 393.9 ± 30.3 | 0.104 |
| Data are expressed as mean ± SEM. | | | |
